# Familial gigantiform cementoma with recurrent ANO5 p.Cys356Tyr mutations: Clinicopathological and genetic study with literature review

**DOI:** 10.1002/mgg3.2277

**Published:** 2023-08-30

**Authors:** Zheng Zhou, Ye Zhang, Lijing Zhu, Yajuan Cui, Yan Gao, Chuan‐Xiang Zhou

**Affiliations:** ^1^ Department of Oral Pathology Peking University School and Hospital of Stomatology Beijing P.R. China; ^2^ National Engineering Laboratory for Digital and Material Technology of Stomatology Peking University School and Hospital of Stomatology Beijing P.R. China

**Keywords:** ANO5, differential diagnosis, familial gigantiform cementoma, gnathodiaphyseal dysplasia

## Abstract

**Background:**

Familial gigantiform cementoma (FGC) is a rare tumor characterized by the early onset of multi‐quadrant fibro‐osseous lesions in the jaws, causing severe maxillofacial deformities. Its clinicopathological features overlap with those of other benign fibro‐osseous lesions. FGC eventually exhibits progressively rapid growth, but no suspected causative gene has been identified.

**Methods:**

In this study, three patients with FGC were recruited, and genomic DNA from the tumor tissue and peripheral blood was extracted for whole‐exome sequencing.

**Results:**

Results showed that all three patients harbored the heterozygous mutation c.1067G > A (p.Cys356Tyr) in the *ANO5* gene. Furthermore, autosomal dominant mutations in *ANO5* at this locus have been identified in patients with gnathodiaphyseal dysplasia (GDD) and are considered a potential causative agent, suggesting a genetic association between FGC and GDD. In addition, multifocal fibrous bone lesions with similar clinical presentations were detected, including five cases of florid cemento‐osseous dysplasia, five cases of polyostotic fibrous dysplasia, and eight cases of juvenile ossifying fibromas; however, none of them harbored mutations in the *ANO5* gene.

**Conclusion:**

Our findings indicate that FGC may be an atypical variant of GDD, providing evidence for the feasibility of *ANO5* gene testing as an auxiliary diagnostic method for complex cases with multiple quadrants.

## Graphical abstract

This study revealed three patients with familial gigantiform cementoma (FGC) carried the heterozygous mutation c.1067G>A (p.Cys356Tyr) in the ANO5 gene which was not found in 8 juvenile ossifying fibromas, 5 polyostotic fibrous dysplasia and 5 florid cemento‐osseous dysplasia. Our findings suggested ANO5 gene testing can be an auxillary diagnostic method for complex fibro‐osseous lesions.

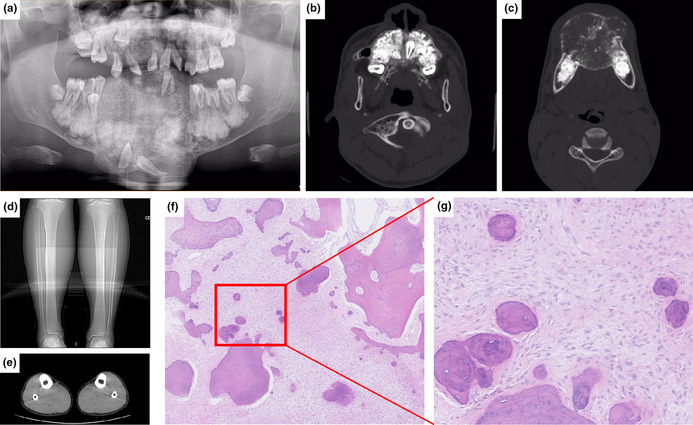

## 1 INTRODUCTION

Familial gigantiform cementoma (FGC), a rare benign tumor often located in the jaw, is characterized by its presence in multiple quadrants, as well as its expansile but well‐defined radiolucent‐radiopaque mixed masses in the mandible and maxilla, which can cause severe maxillofacial deformity (Young et al., 1989). FGC generally occurs in the first two decades of life, and tumor growth can be clinically characterized by three distinctive growth phases: initial onset at 11–13 years of age, rapid expansion between 14 and 16 years of age, and growth suppression around 18–20 years of age (Wang et al., 2015). Histopathologically, it is highly similar to cemento‐ossifying fibroma; cementum‐like calcified deposits are scattered within a fibroblastic stroma, and the proportion of cellular components, size, and the number of calcified deposits are variable (Abdelsayed et al., 2001). Some FGC cases are accompanied by increased bone fragility in the lower extremities and frequent diaphyseal fractures (Ma et al., 2016; Moshref et al., 2008; Rossbach et al., 2005). FCG is a familial autosomal dominant disease; however, some sporadic cases without known heritable features have been described (Eversole et al., 2008). Owing to its rarity, the etiology and molecular pathogenesis of FGC remain unclear.

Gnathodiaphyseal dysplasia (GDD) is a rare bone syndrome characterized by fibrous bone lesions of the jaw, bowing, cortical thickening of long bones, and bone fragility. The syndrome was initially referred to as hereditary gnathodiaphyseal sclerosis. Riminucci et al. have described the histopathological features of jaw lesions, including cementum‐ossifying fibroma, psammomatoid bodies, and abundant mineralization in the vessel wall, and refined the name of this disease entity to gnathodiaphyseal dysplasia, as osteosclerosis is not a feature of the jaw lesions in this syndrome (Riminucci et al., 2001). There is no consensus on the definition of the jaw lesions in GDD, and several different descriptions with different tendencies have been reported; namely, the clinical manifestations of the jaw lesions are similar to those of florid cemento‐osseous dysplasia (FCOD) (Duong et al., 2016), cemento‐ossifying fibromas (Andreeva et al., 2016), or FGC. Therefore, clinical differentiation of GDD from similar diseases requires further research. Based on existing pediatric morbidity analysis, GDD follows an autosomal inheritance pattern. Tsutsumi et al. have indicated that the missense mutation of the *ANO5* gene (also known as TMEM16E) may be a pathogenic mutation of GDD (Tsutsumi et al., 2004).

There has been considerable controversy regarding the nosology of benign fibro‐osseous lesions. FGC was considered a variant of FCOD by the third edition of the World Health Organization (WHO) Classification of Head and Neck Tumors (Barnes et al., 2005) and was stratified as a separate disease entity by the fourth edition (El‐Naggar et al., 2017). Although both FGC and FCOD exhibit microscopic features analogous to those of cemento‐ossifying fibroma, FGC presents with diffuse expansion in multiple quadrants, resulting in remarkable facial disfigurement, whereas FCOD exhibits multi‐quadrant localized areas of expansion involving the apices of the vital teeth. Furthermore, it has been suggested that cemento‐ossifying fibroma may be a part of GDD, and genetic analysis has revealed *ANO5* mutations in some familial FCOD cases (Lv et al., 2019). To date, only a few isolated case reports of FGC and some genetic studies have been published. In the present study, we examined three new cases of FGC using whole‐exome sequencing (WES) and Sanger sequencing and analyzed the related fibro‐osseous lesions to better understand this rare disease and its relationship with FCOD and GDD. Moreover, this study also included a literature review on the limited knowledge of the genetic alterations and clinical features of FGC, GDD, and FCOD.

## 2 MATERIALS AND METHODS

### 2.1 Patients and specimens

A total of 21 cases were analyzed in the present study; three patients with gigantiform cementoma and five patients diagnosed with FCOD with a clinical presentation as that of FGC were enrolled at the Peking University School of Stomatology from 2015 to 2021, and five patients with polyostotic fibrous dysplasia and eight patients with juvenile ossifying fibromas were selected as controls. Age, sex, symptoms, radiological examinations, and other clinical information were obtained from medical records and reviewed retrospectively in detail. Hematoxylin–eosin‐stained sections of formalin‐fixed, paraffin‐embedded (FFPE) tissues were reviewed and evaluated for histopathological findings by three experienced pathologists. This study was approved by the Ethical Review Committee of Peking University School and Hospital of Stomatology. All patients provided informed consent prior to participation.

### 2.2 DNA extraction

Genomic DNA was extracted from FFPE tissues obtained from all 21 cases using the QIAamp DNA Blood & Tissue Kit (Qiagen, Valencia, CA), according to the manufacturer's instructions. For the three gigantiform cementoma cases, both FFPE tissues and peripheral blood were used for genomic DNA extraction, whereas only FFPE tissues were used for the other 18 cases. A NanoDrop8000 (Thermo Fisher Scientific, Waltham, MA, USA) and Qubit2.0 fluorometer (Thermo Fisher Scientific) was used to detect the quality and quantity of genomic DNA.

### 2.3 *ANO5* mutation analysis

All 21 patients were evaluated for potentially pathogenic mutations in exons 7, 11, and 15 of *ANO5* (NCBI gene ID: 203859; NCBI gene reference: NC_000011.9/NM_213599.3). Genomic DNA was amplified in a standard PCR mixture using GoTaq Green Master Mix (Promega, Madison, WI), and Sanger sequencing was performed. The primer sequences and PCR procedure used in this step are shown in detail in Supporting Information Table S1.

### 2.4 WES and bioinformatic analysis

DNA for WES was obtained from the peripheral blood sample and formalin‐fixed gigantiform cementoma tissue of patient #1 before decalcification. For patients #2 and #3, WES was performed on qualified DNA from peripheral blood but not on the DNA extracted from FFPE tissues, owing to its substandard quality.

Genomic DNA was randomly sheared into fragments with an average size of 180–280 bp, and the Agilent SureSelect Human All Exon V5/V6 kit (Agilent Technology, Palo Alto, CA, USA) was used for library preparation. After quality control, the DNA libraries were sequenced on an Illumina HiSeq PE150 platform (Novogene Co, Beijing, China) to obtain paired‐end 150‐bp reads. The raw data obtained by sequencing were subjected to quality control, and qualified clean sequencing reads were aligned to the reference genome (human_B37) using BWA (Li & Durbin, 2009) and Samblaster (Faust & Hall, 2014). SAMtools (Li et al., 2009) was used to perform variant calling and to identify single‐nucleotide variants (SNVs) and insertions/deletions (InDels). Peripheral blood and tumor data were compared using MuTect (Cibulskis et al., 2013) and Strelka (Saunders et al., 2012) to identify somatic SNVs and InDels. Functional annotation of the encoded amino acids for somatic mutations was performed using ANNOVAR software (Wang et al., 2010). Pathway enrichment was completed using the Metascape website (https://metascape.org/) (Zhou et al., 2019). Protein–protein interaction network analysis was conducted using the STRING database (https://cn.string‐db.org/). Protein three‐dimensional structure prediction was performed using AlphaFold 2 TIB server (Tunyasuvunakool et al., 2021).

The parameter ‘Priority’ was designed to evaluate the harmfulness of the SNPs and InDels. A mutation was designated as High Priority if it met the following criteria: location in an exonic or splicing region instead of a repeat genome region (no annotation in the genomicSuperDups and Repeat databases) and a mutation frequency of less than 0.01 according to the 1000 genome database, with no less than one software program (SIFT ≤0.05, Polyphen2_HVAR ≥0.909, Polythen2_HDIV ≥0.957, MutationTaster_pred as ‘D’, and/or CADD_pred >15) predicting the mutation as harmful.

## 3 RESULTS

### 3.1 Basic conditions and clinical characteristics of patients

The clinical information of the three patients with gigantiform cementoma in this study and previously reported cases is presented in Table 1.

**TABLE 1:** The clinical information of three patients and a summary of FGC case reports.

Patients	Age	Sex	Recurrence	Fracture	Familial history	Long bone bowing	Reference
1#	10	M	2	2;left femur	No	No	This study
2#	1	M	1	1;left femur	Yes	No	This study
3#	28	F	1	No	Yes	No	This study
*The present case reports*							
1	6	F	No, but continued	No	Yes, 16 members	No	Young et al (1989).
2	12	F	Yes	No	No	NM	Abdelsayed et al. (2001)
3	9	F	Yes, 2	No	NM	NM	
4	10	F	NM	No	Yes	NM	
5	12	F	NM	Yes	Yes, 4 members	No, but cortical thickening	Ma et al. (2016)
6	17	F	No	Yes	Yes	NM	
7	8	F	NM	Yes	Yes	No, but cortical thickening	
8	6	M	NM	No	Yes	No	
9	24	M	No	Yes	Yes	No	Moshref et al. (2008)
10	25	M	No	Yes	Yes	No	
11	25	M	No	Yes	Yes	No	
12	20	M	No	No	Yes	No	
13	12	M	No	No	No	No	Kumar et al. (2012).
14	6	F	No	No	No	No	Noffke et al. (2012)
15	15 months	F	Yes	No	Yes	No	Shah et al. (2012)
16	2nd decades	M	Yes	No	Yes	NM	
17	34	M	No	No	Yes	No	Şakar (2015)
18	38	F	No	No	Yes	No	
19	11	M	No	Yes	Yes	No	Wang et al. (2015)
20	15	F	NM	NM	NM	NM	Ray (2019)
21	7	M	No	NM	Yes	No	Prasad et al. (2022)

Abbreviation: NM, not mentioned.

**FIGURE 1:**
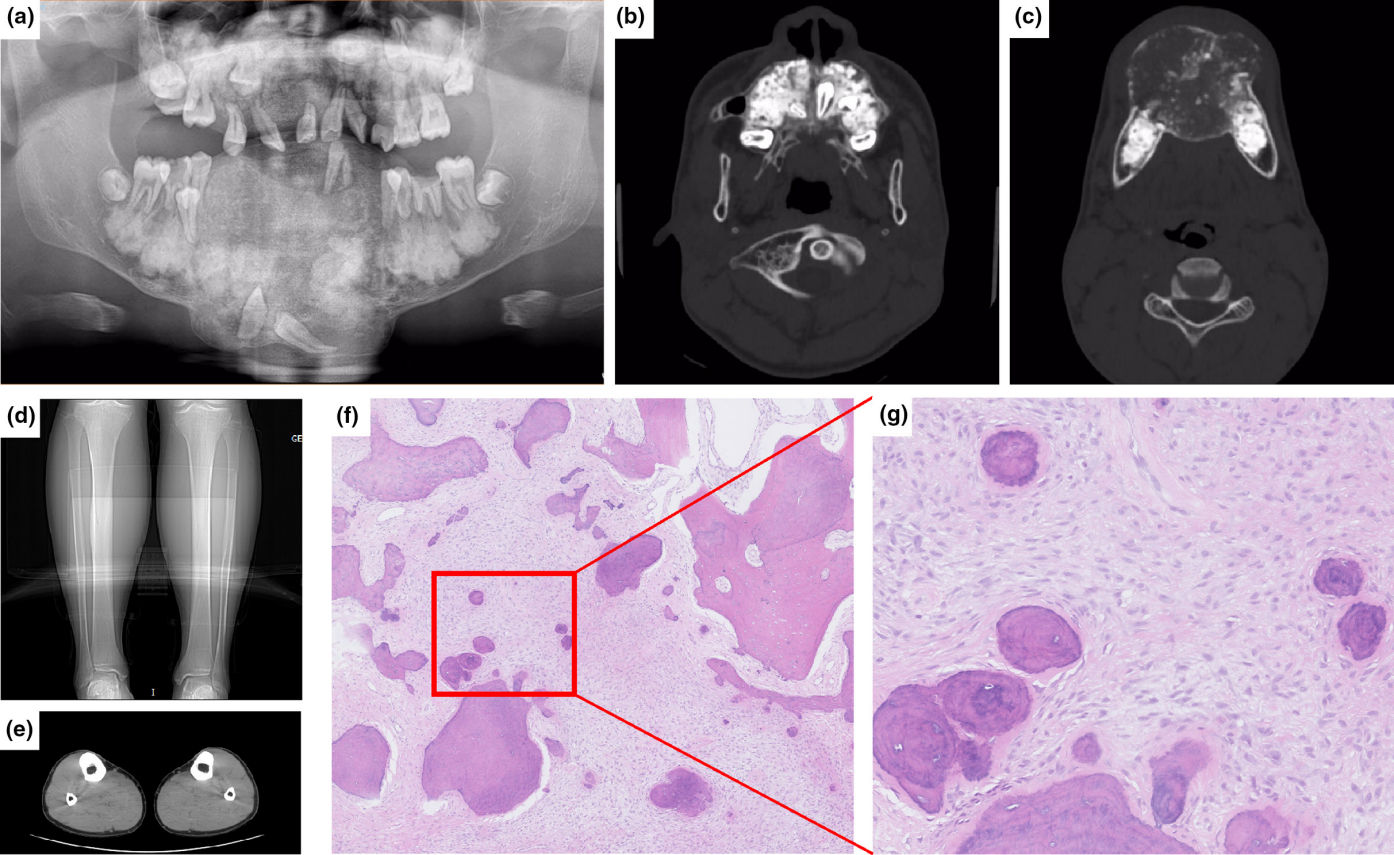
Radiographic and microscopic features of the FGC lesion in Patient #1. (a–c) Panoramic radiographs and computed tomography (CT) images reveal irregular high‐density masses in the tooth‐bearing areas of the maxilla and mandible, composed of radiolucencies and radiopacities in the front of the jaws. (d, e) CT image shows no cortical thickening of the long bones with no curvature on the tibia. (f, g) Histological features of the lesion in a low‐power view (f: 40×) and a high‐power view (g: 200×) show small bones and cement‐like calcified deposits lying within a fibroblastic stroma.

Patient #1 was a 12‐year‐old male. He was referred to our hospital with facial swelling and deformity, mainly in the mandible, which had progressed over 2 years. Curettage and osteoplasty were performed; however, the neoplasm recurred shortly after surgery. Tumor resection was performed two years after the first treatment; however, the tumor continued to grow, and facial deformation was still evident after the second surgery. Panoramic radiography revealed irregular high‐density masses in the tooth‐bearing areas of the maxilla and mandible. The lesion was composed of radiolucencies and radiopacities in the front of the mandible containing two impacted teeth, and the dentition was displaced owing to pressure. The patient had a history of bone fractures and suffered from a fracture in the left femur while running during adolescence. No pathological bowing of the long bones was noted throughout the body, and computed tomography (CT) showed no cortical thickening of the long bones (Figure 1). There was a slight increase in alkaline phosphatase levels in the blood before the last surgery. None of the other family members had similar symptoms.

**FIGURE 2:**
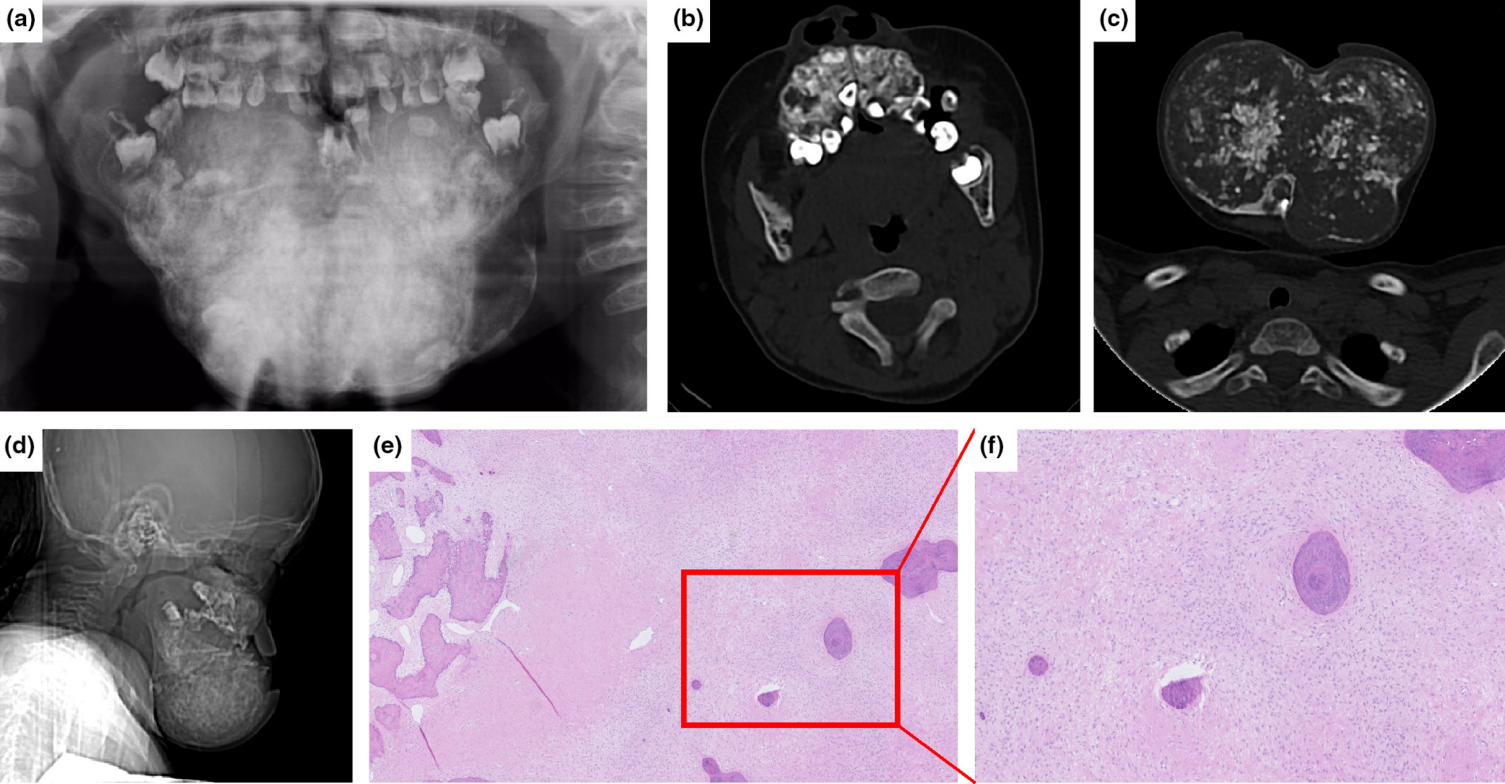
Radiographic and microscopic features of the FGC lesion in Patient #2. (a–c) Panoramic radiographs and computed tomography (CT) images show irregular masses composed of radiolucencies and radiopacities in multi‐quadrants. (d) CT image shows the severe expansion of the mandible resulting in difficulty for the patient in closing the mouth. (e, f) Histological features of the lesion in a low‐power view (e: 40×) and a high‐power view (f: 200×) show small bones and cement‐like calcified deposits lying within a fibroblastic stroma.

Patient #2 was a 3‐year‐old boy who presented with swelling in the mandible and maxilla. The chief concern was facial deformity caused by an expansive tumor‐like lesion at 6 months of age. A grain‐sized white protuberant particle appeared on the posterior mandible during the early stage, and a gradual enlargement involving four quadrants resulted in obvious facial disfigurement. Curettage was initially attempted; however, the tumor recurred with an accelerated growth rate, accompanied by tooth compression and displacement. Mandibular segmental osteotomy and implantation of the reconstruction plate were performed two years after the first surgery. Radiographic examination revealed a multi‐quadrant and expansile lesion on both sides of the maxilla and mandible, and the main lesion in the posterior mandible was composed of radiolucent and radiopaque masses (Figure 2). He experienced bone fractures twice in the left and right femurs; however, no other pathological findings of the long bone were observed. He was the son of patient #3.

**FIGURE 3:**
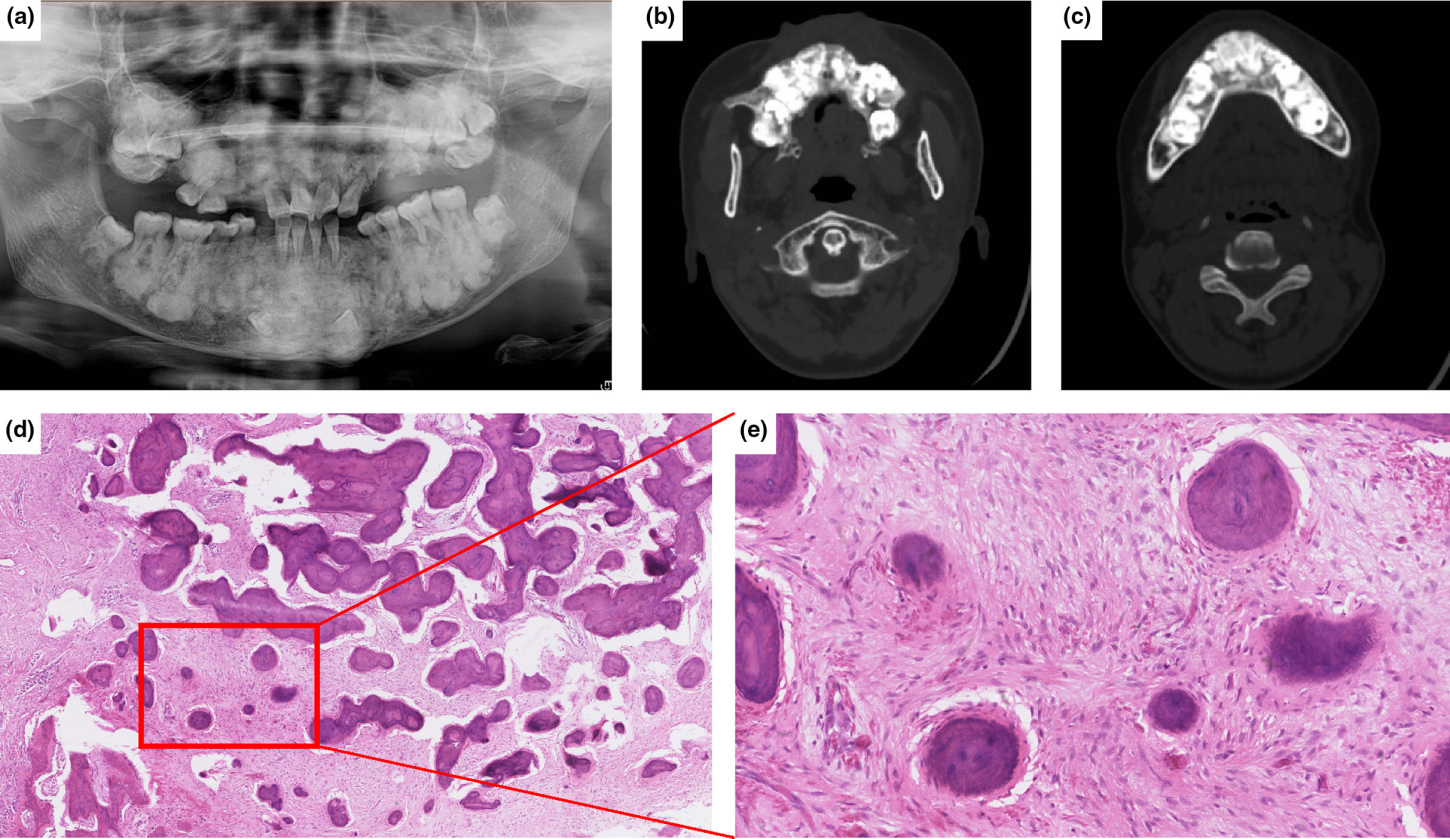
Radiographic and microscopic features of the FGC lesion in Patient #3. (a–c) Panoramic radiographs and computed tomography (CT) images show irregular high‐density masses in the tooth‐bearing area of the maxilla and mandible. (d, e) Histological features of the lesion in a low‐power view (d: 40×) and a high‐power view (e: 200×) show small, round, and disconnected bones lying within a fibroblastic stroma.

Patient #3 was a 28‐year‐old woman who was the mother of patient #2. She was referred to our hospital with a complaint of swelling and pain on the left side of her face after exertion. Panoramic radiography displayed multiple radiopaque irregular masses in the tooth‐bearing area of the bilateral upper and lower jaws with space infection. In this patient, most of the maxilla lost its normal trabecular structure, the mandibular cortex was compressed and thinned, and the dentition was compressed and displaced (Figure 3). Her alkaline phosphatase and creatine kinase levels were normal, and there was no history of fracture. None of the other family members had symptoms similar to those of patients #2 and #3.

### 3.2 *ANO5* mutation and bioinformatics analysis

**FIGURE 4:**
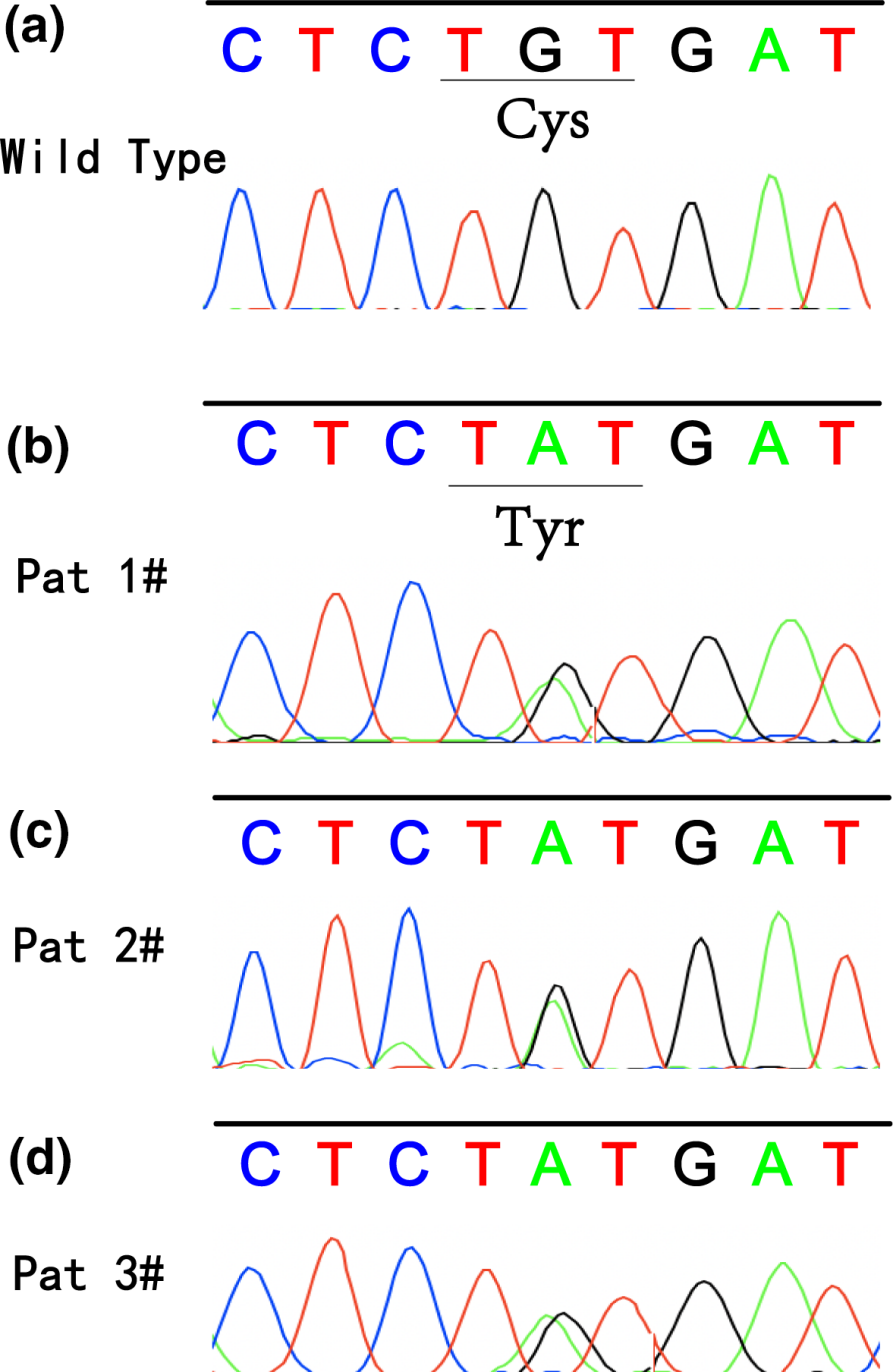
*ANO5* Mutational analysis. (a) The wild‐type sequence of *ANO5* at the Cys356 codon. (b–d) The missense mutation at the Cys356 codon.

DNA was extracted from the tumor samples of all 21 patients after surgery, and mutations in exons 11 and 15 of *ANO5* were detected. The c.1067 G>T (p.Cys356Tyr) heterozygous missense mutation was detected using genomic DNA from both tumor samples and peripheral blood samples of patients #1, #2, and #3 with gigantiform cementoma (Figure 4a–d). C356 was found to be highly conserved across species (Supporting Information Figure S1), and no other mutations were present in *ANO5*. In addition, this mutation in *ANO5* was confirmed to be absent in the control group, including five patients with FCOD, five patients with polyostotic fibrous dysplasia, and eight patients with juvenile ossifying fibromas.

We predicted the type of the detected mutation using several software programs: SIFT predicted it to be “deleterious, with a score of 0”; polythen2_HVAR and polythen2_HDIV predicted it to be “probably damaging,” with scores of 0.989 and 1, respectively; and MutationTaster predicted it to be “Disease_causing,” with a score of 1, indicating that the results are reliable. The CADD score was 28, indicating that the mutation pathogenicity was approximately 0.1% in the top SNVs, and the pathogenicity was significant.

**FIGURE 5:**
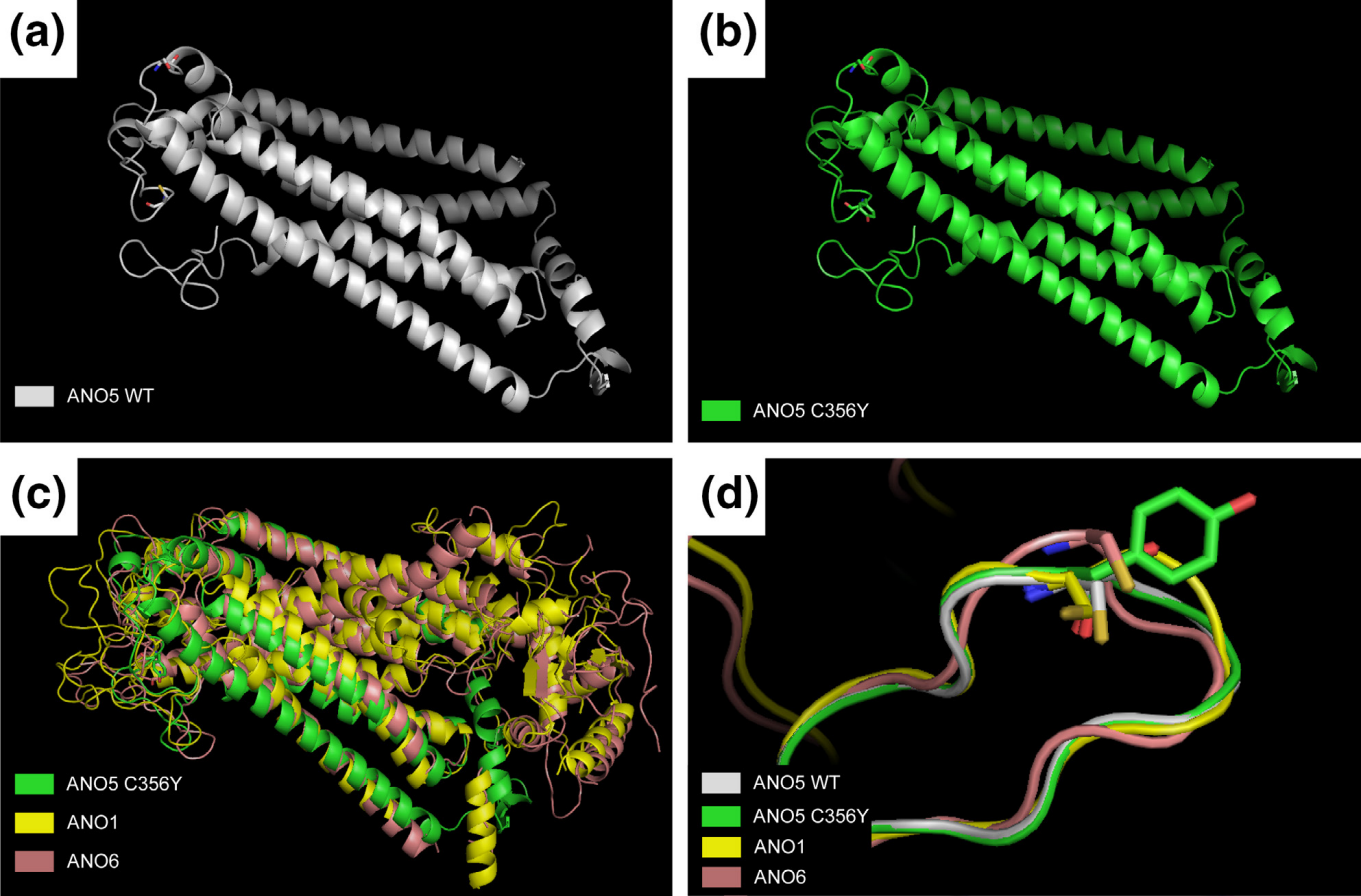
Three‐dimensional structural prediction of the *ANO5* gene. (a) 3D structure of the wild‐type ANO5 protein simulated through the AlphaFold 2 TIB server. (b) 3D structure of the p. C356Y‐mutant ANO5 protein simulated under an RMSD value of 0.256 with the wild‐type ANO5 protein. (c) Wild‐type and C356Y‐mutant ANO5 proteins aligned with fungal homologs ANO1 and ANO6. (d) Residues Lys847, Phe848, and Leu 849 in ANO5 (C356Y) created a loop, whereas the equivalent residues in the wild‐type ANO5 formed a helix structure.

To further predict the effects of this mutation on the structure and function of the ANO5 protein, we constructed molecular models of the wild‐type and p.Cys356Tyr mutant ANO5 protein using the AlphaFold 2 TIB server. The published crystal structures of the fungal homologs ANO1 and ANO6 revealed 8–10 transmembrane domains, and the predicted structure of wild‐type ANO5 was consistent with the putative structure of the anoctamin family members. Based on AlphaFold 2 analysis, the structure of the ANO5 C356Y mutant did not markedly deviate from the wild‐type structure (RMSD: 0.256); however, these deviations were evident in the region around the location of the mutated residue. Residues Lys847, Phe848, and Leu849 were estimated to form a helix in the wild‐type, whereas they were predicted to create a loop in the mutant p.Cys356Tyr ANO5 protein, which may affect its structure and function (Figure 5a–d). The *ANO5* mutation loci and types reported to date for GDD and their related lesions are detailed in Supporting Information Table S2.

### 3.3 SNVs and InDels revealed by WES

The fixation, storage, and decalcification processes of FFPE tissues may damage DNA, resulting in a decrease in DNA library quality. For the DNA extracted from FFPE tumor tissue, only that from patient #1 met the WES requirements; therefore, genomic DNA from both peripheral blood samples and tumor tissue of patient #1 was available for WES analysis; however, for patients #2 and #3, only the genomic DNA from the peripheral blood samples was subjected to WES.

**FIGURE 6:**
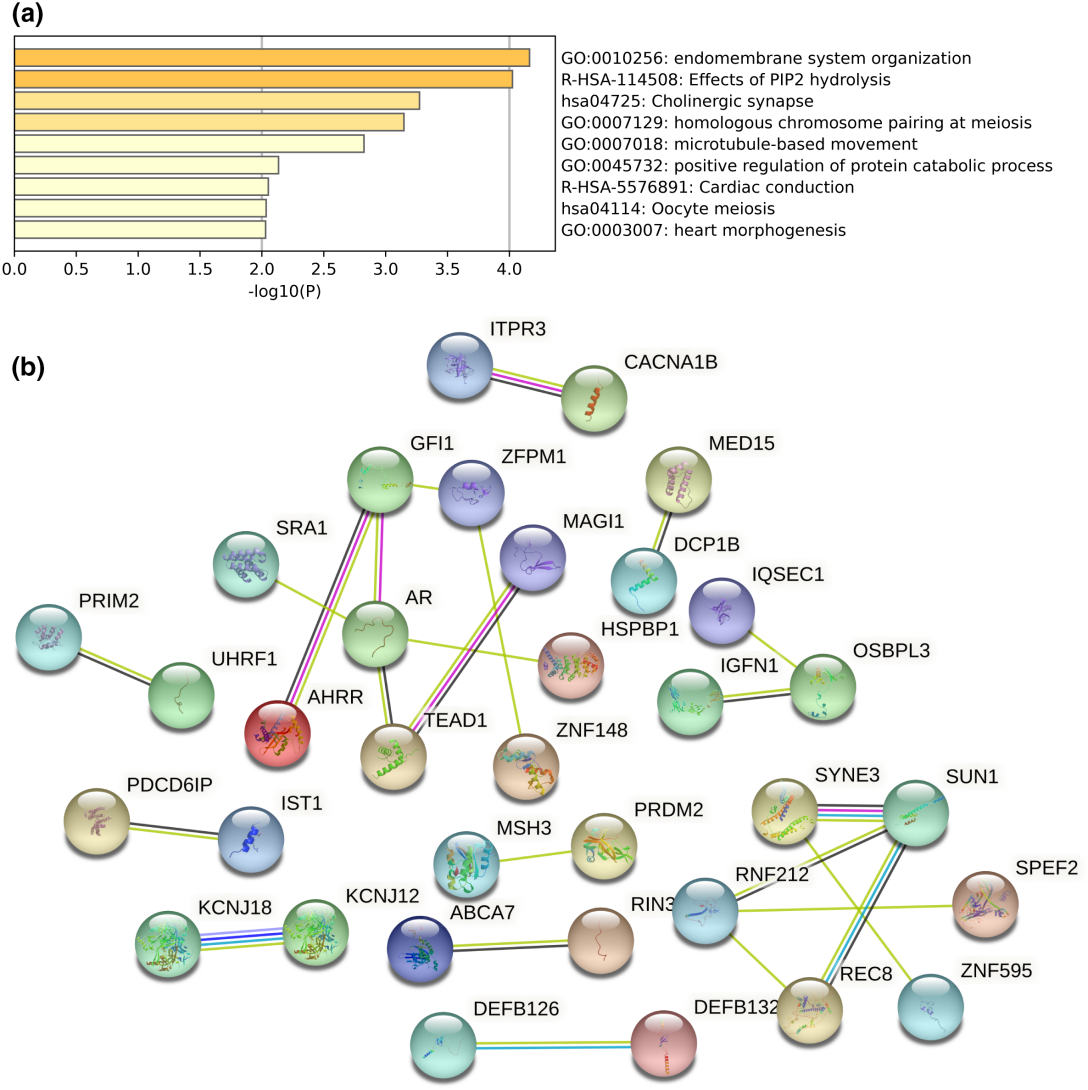
Gene‐enrichment and protein–protein interaction analyses. (a) The enrichment pathways of ‘H’ priority‐mutated genes and (b) protein–protein interactions among ‘H’ priority genes.

In total, 643, 614, and 595 high‐priority SNVs and 154, 127, and 121 high‐priority InDels were detected in the tissues of patients #1, #2, and #3, respectively. Detailed information on these SNVs and InDels is listed in Supporting Information Tables S3 and S4, respectively. By counting both the SNPs and InDels of a gene as alterations, we found that 103 genes were simultaneously altered in the three patients. Pathway enrichment analysis was performed on this group of genes ranked according to their significance from high to low, and the results showed that the phosphatidylinositol signaling system, cholinergic synapse, homologous chromosome pairing at meiosis, microtubule‐based movement, and the positive regulation of protein catabolic process were enriched in the top GO terms, as shown in Figure 6a. The results of the protein–protein interaction analysis are shown in Figure 6b.

## 4 DISCUSSION

In the present study, detailed clinical manifestations, as well as pathologic and radiographic data, were retrieved from three patients with FGC. According to the literature, not all patients with FGC have a family history, and some cases are sporadic (Abdelsayed et al., 2001; Kumar et al., 2012; Noffke et al., 2012). In the present study, one patient (patient #1) was sporadic, whereas the other two patients were from the same family (patient #3 was the mother of patient #2). The age of onset ranges from 1 to 38 years, and it is most common during adolescence, followed by early childhood (Prasad et al., 2022; Şakar et al., 2015; Shah et al., 2012; Wang et al., 2015), with no sex tendency. Owing to its nature, the recurrence rate of FGC is high, and incomplete resection or conservative curettage often leads to more rapid progression of the remaining portion, which does not recur after complete resection (Wang et al., 2017). In the present study, the three cases were consistent with FGC in terms of age, the severity of jaw expansion, site of onset, concomitant symptoms, treatment strategy, and recurrence, with no other bones affected. Therefore, FGC was diagnosed, although one case had no family history.

FGC is a rare fibro‐osseous lesion that is not well defined. Owing to its rarity, only a few isolated cases have been reported, and genetic studies are currently unavailable. GDD, an autosomal dominant generalized skeletal syndrome, is characterized by fibro‐osseous lesions of the jaw, combined with bowing and fragility of the long bones (Riminucci et al., 2001). The gnathic lesions in GDD have been variably described, and some cases exhibited a typical FGC phenotype and analogous pathologic features in jaw lesions. Furthermore, the treatment and prognosis of jaw lesions in GDD are analogous to those described for FGC (Andreeva et al., 2016; Duong et al., 2016), which is characterized by multiple, rapid local recurrences after surgery. However, whether FGC and GDD are genetically related remains unclear.

Recently, a missense mutation in *ANO5* (also known as TMEM16E) was identified in GDD and was considered a suspected causative mutation for GDD (Duong et al., 2016; Jin et al., 2017; Marconi et al., 2013; Mizuta et al., 2007; Otaify et al., 2018; Tsutsumi et al., 2004; Zeng et al., 2019). In the present study, we identified a heterozygous missense mutation, c.1067 G>T(p.Cys356Tyr), in *ANO5* in all three patients with FGC based on WES and confirmed it by Sanger sequencing. No patients in the control group, including five patients with FCOD, five patients with polyostotic fibrous dysplasia, and eight patients with juvenile ossifying fibromas, carried mutated *ANO5*, suggesting that this type of mutation is uncommon in multifocal/multiquadrant fibro‐osseous lesions. To the best of our knowledge, this is the first genetic study on FGC, and this specific mutation at the same locus has been identified as a causative mutation in patients with GDD, indicating that FGC is genetically correlated with GDD. Owing to the similarities in the clinicopathological features and genetic alterations between GDD and FGC, we suggest that FGC may be an atypical subtype of GDD, which may have no systemic symptoms, classic long bone curvature, and cortical thickening, with fibro‐osseous lesions of the jawbone as the only obvious symptoms. Other studies have also reported atypical cases of GDD where the patient's age of onset, the extent of expansion, and pathological and imaging manifestations also overlap with those of FGC (Andreeva et al., 2016; Zeng et al., 2019).

*ANO5* is also referred to as TMEM16E or GDD1. Mutations at other *ANO5* loci can cause muscle diseases, such as proximal limb‐girdle muscular dystrophy and distal non‐dysferlin Miyoshi myopathy (Bolduc et al., 2010; Shaibani et al., 2021). However, there is no overlap between mutant loci for bone and muscle diseases; mutations that cause bone lesions do not cause muscle lesions in patients, and vice versa (Marconi et al., 2013). The pathogenic mechanism of *ANO5* site‐specific mutations has not yet been clearly explained, and some gene‐edited animal disease models have successfully replicated GDD syndrome‐like manifestations, including long bone bowing, thickened bone cortex, jaw masses, and increased bone fragility (Li et al., 2021; Wang et al., 2019). However, in mouse models, microscopic analysis revealed increased bone mineralization in the jaw lesions but not the microscopic features of fibrous‐osseous lesions generally detected in patients with GDD. Previous in vitro experiments have reported that mutations in *ANO5* may play a role in osteoblast differentiation (Kim et al., 2019). However, whether fibrous‐osseous lesions in patients with GDD require additional causative factors other than *ANO5* mutations should be validated in further studies.

As both SNVs and InDels may have deleterious effects on the physiological functions of genes, we considered a gene as potentially pathogenic when it had a high priority for SNVs and InDels. Based on WES analysis, we extracted 103 potentially pathogenic genes, including *ANO5*, that were present in all three patients. These genes were enriched in pathways that regulate a wide range of physiological processes, such as endomembrane system organization and positive regulation of protein catabolic process. In the potential pathogenic gene set, we extracted the genes simultaneously predicted to be deleterious by SIFT, Polythen2, and MutationTaster. Among these genes, PED4FIP and MUC20 were noted as frequently mutated in malignant tumors and play a role in tissue damage repair and tumor growth through activation of the HGF‐MET pathway, and some evidence suggests that their mutation and expression levels correlate with prognosis (Chen et al., 2013, 2016). Furthermore, MET‐sustained activation in tumors is a late event that maintains the malignancy of transformed cells by conveying proliferative, pro‐migratory, and anti‐apoptosis signals (Chen et al., 2018; Trusolino et al., 2010). *CACNA1B*, a gene with similar effects to those of the ANOs family, functions as a voltage‐sensitive calcium channel (Williams et al., 1992). Both its mutation and the *ANO3* mutation are associated with myoclonus‐dystonia (Domingo et al., 2016).Although both ITPR3 and CACNA1B act on cytoplasmic calcium ion concentration, the specific effects of these mutations have not been confirmed in vivo, warranting further study.

There is considerable controversy regarding the nosology of benign fibro‐osseous lesions. FGC was once referred to as a variant of FCOD (Melrose et al., 1976; Waldron et al., 1975; Wolf et al., 1989) and has also been suggested to be a variant of ossifying fibroma, as the progressive growth of FGC suggests a neoplastic process. Familial FCOD (FFCOD) is the fourth subtype introduced in the 2022 WHO classification (Vered & Wright, 2022), which raises a more challenging differential diagnosis with FGC. Compared with conventional FCOD, FFCOD presents an earlier onset, usually tends to cause considerable jawbone expansion, and does not favor a specific sex or ethnic group. Moreover, a missense mutation (p.C356W) in the *ANO5* gene has been identified in one family with FFCOD (Lv et al., 2019), whereas our genetic analysis revealed a missense mutation (p.C356Y) in the *ANO5* gene in all three patients with FGC. Therefore, more cases and further studies are required to identify whether FFCOD and FGC represent different spectrums of disease progression.

In summary, benign fibro‐osseous lesions are a group of diseases with overlapping clinicopathological features, and it is difficult to obtain a correct diagnosis in some sophisticated cases. This study demonstrated the feasibility of *ANO5* gene testing as an auxiliary diagnostic method for complex cases with multiple quadrants. Our findings suggest that FGC may be an atypical variant of GDD based on genetic association. Patients with early onset should be alerted of the possibility of rapid growth and undergo a timely complete resection of the mass.

## AUTHOR CONTRIBUTIONS

Chuan‐Xiang Zhou performed the study design and concept. Zheng Zhou and Ye Zhang performed the methodology and the writing, review, and revision of the paper. Zheng Zhou, Yajuan Cui and Lijing Zhu performed the data collection. Yan Gao provided technical and material support. Zheng Zhou and Chuan‐Xiang Zhou performed data analysis and interpretation.

## FUNDING INFORMATION

This research was supported by research grants from the National Natural Science Foundation of China (82103061).

## CONFLICT OF INTEREST STATEMENT

None declared.

## ETHICS APPROVAL AND CONSENT TO PARTICIPATE

This study was approved by the regional Ethical Review Board of Peking University School and Hospital of Stomatology and conducted in accordance with the Declaration of Helsinki. All patients provided written informed consent.

## Supporting information


Figure S1.

Supplementary Table 1.

Supplementary Table 2.

Supplementary Table 3.

Supplementary Table 4.

## Data Availability

The data of this study are available from the corresponding author upon reasonable request.

## References

[mgg32277-bib-0001] Abdelsayed, R. A. , Eversole, L. R. , Singh, B. S. , & Scarbrough, F. E. (2001). Gigantiform cementoma: Clinicopathologic presentation of 3 cases. Oral Surgery, Oral Medicine, Oral Pathology, Oral Radiology, and Endodontics, 91, 438–444. 11312460 10.1067/moe.2001.113108

[mgg32277-bib-0002] Andreeva, T. V. , Tyazhelova, T. V. , Rykalina, V. N. , Gusev, F. E. , Goltsov, A. Y. , Zolotareva, O. I. , Aliseichik, M. P. , Borodina, T. A. , Grigorenko, A. P. , Reshetov, D. A. , Ginter, E. K. , Amelina, S. S. , Zinchenko, R. A. , & Rogaev, E. I. (2016). Whole exome sequencing links dental tumor to an autosomal‐dominant mutation in ANO5 gene associated with gnathodiaphyseal dysplasia and muscle dystrophies. Scientific Reports, 6, 26440. 27216912 10.1038/srep26440 PMC4877638

[mgg32277-bib-0003] Barnes, L. , Eveson, J. W. , Reichart, P. , & Sidransky, D. (2005). WHO classification of head and neck tumours (3rd ed.). Lyon.

[mgg32277-bib-0004] Bolduc, V. , Marlow, G. , Boycott, K. M. , Saleki, K. , Inoue, H. , Kroon, J. , Itakura, M. , Robitaille, Y. , Parent, L. , Baas, F. , Mizuta, K. , Kamata, N. , Richard, I. , Linssen, W. H. J. P. , Mahjneh, I. , de Visser, M. , Bashir, R. , & Brais, B. (2010). Recessive mutations in the putative calcium‐activated chloride channel anoctamin 5 cause proximal LGMD2L and distal MMD3 muscular dystrophies. American Journal of Human Genetics, 86, 213–221. 20096397 10.1016/j.ajhg.2009.12.013 PMC2820170

[mgg32277-bib-0005] Chen, C. H. , Shyu, M. K. , Wang, S. W. , Chou, C. H. , Huang, M. J. , Lin, T. C. , Chen, S. T. , Lin, H. H. , & Huang, M. C. (2016). MUC20 promotes aggressive phenotypes of epithelial ovarian cancer cells via activation of the integrin β1 pathway. Gynecologic Oncology, 140, 131–137. 26616226 10.1016/j.ygyno.2015.11.025

[mgg32277-bib-0006] Chen, C. H. , Wang, S. W. , Chen, C. W. , Huang, M. R. , Hung, J. S. , Huang, H. C. , Lin, H. H. , Chen, R. J. , Shyu, M. K. , & Huang, M. C. (2013). MUC20 overexpression predicts poor prognosis and enhances EGF‐induced malignant phenotypes via activation of the EGFR‐STAT3 pathway in endometrial cancer. Gynecologic Oncology, 128, 560–567. 23262208 10.1016/j.ygyno.2012.12.012

[mgg32277-bib-0007] Chen, S. T. , Kuo, T. C. , Liao, Y. Y. , Lin, M. C. , Tien, Y. W. , & Huang, M. C. (2018). Silencing of MUC20 suppresses the malignant character of pancreatic ductal adenocarcinoma cells through inhibition of the HGF/MET pathway. Oncogene, 37, 6041–6053. 29993037 10.1038/s41388-018-0403-0 PMC6237765

[mgg32277-bib-0008] Cibulskis, K. , Lawrence, M. S. , Carter, S. L. , Sivachenko, A. , Jaffe, D. , Sougnez, C. , Gabriel, S. , Meyerson, M. , Lander, E. S. , & Getz, G. (2013). Sensitive detection of somatic point mutations in impure and heterogeneous cancer samples. Nature Biotechnology, 31, 213–219. 10.1038/nbt.2514 PMC3833702 23396013

[mgg32277-bib-0009] Domingo, A. , Erro, R. , & Lohmann, K. (2016). Novel dystonia genes: Clues on disease mechanisms and the complexities of high‐throughput sequencing. Movement Disorders, 31, 471–477. 26991507 10.1002/mds.26600

[mgg32277-bib-0010] Duong, H. A. , Le, K. T. , Soulema, A. L. , Yueh, R. H. , Scheuner, M. T. , Holick, M. F. , Christensen, R. , Tajima, T. L. , Leung, A. M. , & Mallya, S. M. (2016). Gnathodiaphyseal dysplasia: Report of a family with a novel mutation of the ANO5 gene. Oral Surgery, Oral Medicine, Oral Pathology, Oral Radiology, 121, e123–e128. 27068316 10.1016/j.oooo.2016.01.014 PMC4830924

[mgg32277-bib-0011] El‐Naggar, A. K. , John, K. C. , Grandis, J. R. , Takata, T. , & Slootweg, P. T. (2017). WHO classification of head and neck tumours (4th ed.). Lyon.

[mgg32277-bib-0012] Eversole, R. , Su, L. , & ElMofty, S. (2008). Benign fibro‐osseous lesions of the craniofacial complex. A Review. Head and Neck Pathology, 2, 177–202. 20614314 10.1007/s12105-008-0057-2 PMC2807558

[mgg32277-bib-0013] Faust, G. G. , & Hall, I. M. (2014). SAMBLASTER: Fast duplicate marking and structural variant read extraction. Bioinformatics, 30, 2503–2505. 24812344 10.1093/bioinformatics/btu314 PMC4147885

[mgg32277-bib-0014] Jin, L. , Liu, Y. , Sun, F. , Collins, M. T. , Blackwell, K. , Woo, A. S. , Reichenberger, E. J. , & Hu, Y. (2017). Three novel ANO5 missense mutations in Caucasian and Chinese families and sporadic cases with gnathodiaphyseal dysplasia. Scientific Reports, 7, 40935. 28176803 10.1038/srep40935 PMC5296836

[mgg32277-bib-0015] Kim, J. H. , Kim, K. , Kim, I. , Seong, S. , Kim, S. W. , & Kim, N. (2019). Role of anoctamin 5, a gene associated with gnathodiaphyseal dysplasia, in osteoblast and osteoclast differentiation. Bone, 120, 432–438. 30557634 10.1016/j.bone.2018.12.010

[mgg32277-bib-0016] Kumar, V. V. , Ebenezer, S. , Narayan, T. V. , & Wagner, W. (2012). Clinicopathologic conference: Multiquadrant expansile fibro‐osseous lesion in a juvenile. Oral Surgery, Oral Medicine, Oral Pathology, Oral Radiology, 113, 286–292. 22669141 10.1016/j.tripleo.2011.08.021

[mgg32277-bib-0017] Li, H. , & Durbin, R. (2009). Fast and accurate short read alignment with burrows‐wheeler transform. Bioinformatics, 25, 1754–1760. 19451168 10.1093/bioinformatics/btp324 PMC2705234

[mgg32277-bib-0018] Li, H. , Handsaker, B. , Wysoker, A. , Fennell, T. , Ruan, J. , Homer, N. , Marth, G. , Abecasis, G. , Durbin, R. , & 1000 Genome Project Data Processing Subgroup . (2009). The sequence alignment/map format and SAMtools. Bioinformatics, 25, 2078–2079. 19505943 10.1093/bioinformatics/btp352 PMC2723002

[mgg32277-bib-0019] Li, H. , Wang, X. , Chen, E. , Liu, X. , Ma, X. , Miao, C. , Tian, Z. , Dong, R. , & Hu, Y. (2021). Introduction of a Cys360Tyr mutation in ANO5 creates a mouse model for gnathodiaphyseal dysplasia. Journal of Bone and Mineral Research, 37, 515–530. 34841576 10.1002/jbmr.4481

[mgg32277-bib-0020] Lv, M. , You, G. , Wang, J. , Fu, Q. , Gupta, A. , Li, J. , & Sun, J. (2019). Identification of a novel ANO5 missense mutation in a Chinese family with familial florid osseous dysplasia. Journal of Human Genetics, 64, 599–607. 30996299 10.1038/s10038-019-0601-9

[mgg32277-bib-0021] Ma, C. , Wang, H. , He, G. , & Qin, X. (2016). Familial gigantiform cementoma: Case report of an unusual clinical manifestation and possible mechanism related to “calcium steal disorder”. Medicine (Baltimore), 95, e2956. 26945411 10.1097/MD.0000000000002956 PMC4782895

[mgg32277-bib-0022] Marconi, C. , Brunamonti Binello, P. , Badiali, G. , Caci, E. , Cusano, R. , Garibaldi, J. , Pippucci, T. , Merlini, A. , Marchetti, C. , Rhoden, K. J. , Galietta, L. J. V. , Lalatta, F. , Balbi, P. , & Seri, M. (2013). A novel missense mutation in ANO5/TMEM16E is causative for gnathodiaphyseal dyplasia in a large Italian pedigree. European Journal of Human Genetics, 21, 613–619. 23047743 10.1038/ejhg.2012.224 PMC3658193

[mgg32277-bib-0023] Melrose, R. J. , Abrams, A. M. , & Mills, B. G. (1976). Florid osseous dysplasia. A clinical‐pathologic study of thirty‐four cases. Oral Surgery, Oral Medicine, and Oral Pathology, 41, 62–82. 1061039 10.1016/0030-4220(76)90254-1

[mgg32277-bib-0024] Mizuta, K. , Tsutsumi, S. , Inoue, H. , Sakamoto, Y. , Miyatake, K. , Miyawaki, K. , Noji, S. , Kamata, N. , & Itakura, M. (2007). Molecular characterization of GDD1/TMEM16E, the gene product responsible for autosomal dominant gnathodiaphyseal dysplasia. Biochemical and Biophysical Research Communications, 357, 126–132. 17418107 10.1016/j.bbrc.2007.03.108

[mgg32277-bib-0025] Moshref, M. , Khojasteh, A. , Kazemi, B. , Roudsari, M. V. , Varshowsaz, M. , & Eslami, B. (2008). Autosomal dominant gigantiform cementoma associated with bone fractures. American Journal of Medical Genetics. Part A, 146a, 644–648. 18247420 10.1002/ajmg.a.32171

[mgg32277-bib-0026] Noffke, C. E. , Ngwenya, S. P. , Nzima, N. , Raubenheimer, E. J. , & Rakgwale, N. B. (2012). Gigantiform cementoma in a child. Dento Maxillo Facial Radiology, 41, 264–266. 22378756 10.1259/dmfr/13435626 PMC3520290

[mgg32277-bib-0027] Otaify, G. A. , Whyte, M. P. , Gottesman, G. S. , McAlister, W. H. , Eric Gordon, J. , Hollander, A. , Andrews, M. V. , el‐Mofty, S. K. , Chen, W. S. , Veis, D. V. , Stolina, M. , Woo, A. S. , Katsonis, P. , Lichtarge, O. , Zhang, F. , & Shinawi, M. (2018). Gnathodiaphyseal dysplasia: Severe atypical presentation with novel heterozygous mutation of the anoctamin gene (ANO5). Bone, 107, 161–171. 29175271 10.1016/j.bone.2017.11.012 PMC5987759

[mgg32277-bib-0028] Prasad, C. , Kumar, K. A. , Balaji, J. , Arulmozhi, M. , Jayanandhini, S. , & Priyadharshini, R. (2022). A family of familial gigantiform cementoma: Clinical study. Journal of Oral and Maxillofacial Surgery, 21, 44–50. 10.1007/s12663-021-01515-2 PMC8934829 35400930

[mgg32277-bib-0029] Ray, A. , Frey, H. M. , & Carron, J. D. (2019). An Unusual Case of Lacrimal Duct Obstruction in a Teenager. JAMA Otolaryngol Head Neck Surgery, 145(4), 381–382. 10.1001/jamaoto.2018.4264 30789663

[mgg32277-bib-0030] Riminucci, M. , Collins, M. T. , Corsi, A. , Boyde, A. , Murphey, M. D. , Wientroub, S. , Kuznetsov, S. A. , Cherman, N. , Robey, P. G. , & Bianco, P. (2001). Gnathodiaphyseal dysplasia: A syndrome of fibro‐osseous lesions of jawbones, bone fragility, and long bone bowing. Journal of Bone and Mineral Research, 16, 1710–1718. 11547842 10.1359/jbmr.2001.16.9.1710

[mgg32277-bib-0031] Rossbach, H. C. , Letson, D. , Lacson, A. , Ruas, E. , & Salazar, P. (2005). Familial gigantiform cementoma with brittle bone disease, pathologic fractures, and osteosarcoma: A possible explanation of an ancient mystery. Pediatric Blood & Cancer, 44, 390–396. 15602717 10.1002/pbc.20253

[mgg32277-bib-0032] Şakar, O. , Aren, G. , Mumcu, Z. , Ünalan, F. , Aksakallı, N. , & Tolgay, C. G. (2015). Familial gigantiform cementoma with Ehlers—Danlos syndrome: A report of 2 cases. Journal of Advanced Prosthodontics, 7(2), 178–182. 25932318 10.4047/jap.2015.7.2.178 PMC4414950

[mgg32277-bib-0033] Saunders, C. T. , Wong, W. S. , Swamy, S. , et al. (2012). Strelka: Accurate somatic small‐variant calling from sequenced tumor‐normal sample pairs. Bioinformatics, 28, 1811–1817. 22581179 10.1093/bioinformatics/bts271

[mgg32277-bib-0034] Shah, S. , Huh, K. H. , Yi, W. J. , Heo, M. S. , Lee, S. S. , & Choi, S. C. (2012). Follow‐up CT findings of recurrent familial gigantiform cementoma of a female child. Skeletal Radiology, 41, 341–346. 21830054 10.1007/s00256-011-1245-9

[mgg32277-bib-0035] Shaibani, A. , Khan, S. , & Shinawi, M. (2021). Autosomal dominant ANO5‐related disorder associated with myopathy and gnathodiaphyseal dysplasia. Neurol Genet., 7, e612. 34291158 10.1212/NXG.0000000000000612 PMC8290902

[mgg32277-bib-0036] Trusolino, L. , Bertotti, A. , & Comoglio, P. M. (2010). MET signalling: Principles and functions in development, organ regeneration and cancer. Nature Reviews. Molecular Cell Biology, 11, 834–848. 21102609 10.1038/nrm3012

[mgg32277-bib-0037] Tsutsumi, S. , Kamata, N. , Vokes, T. J. , Maruoka, Y. , Nakakuki, K. , Enomoto, S. , Omura, K. , Amagasa, T. , Nagayama, M. , Saito‐Ohara, F. , Inazawa, J. , Moritani, M. , Yamaoka, T. , Inoue, H. , & Itakura, M. (2004). The novel gene encoding a putative transmembrane protein is mutated in gnathodiaphyseal dysplasia (GDD). American Journal of Human Genetics, 74, 1255–1261. 15124103 10.1086/421527 PMC1182089

[mgg32277-bib-0038] Tunyasuvunakool, K. , Adler, J. , Wu, Z. , Green, T. , Zielinski, M. , Žídek, A. , Bridgland, A. , Cowie, A. , Meyer, C. , Laydon, A. , Velankar, S. , Kleywegt, G. J. , Bateman, A. , Evans, R. , Pritzel, A. , Figurnov, M. , Ronneberger, O. , Bates, R. , Kohl, S. A. A. , … Hassabis, D. (2021). Highly accurate protein structure prediction for the human proteome. Nature, 596, 590–596. 34293799 10.1038/s41586-021-03828-1 PMC8387240

[mgg32277-bib-0039] Vered, M. , & Wright, J. M. (2022). Update from the 5th edition of the World Health Organization classification of head and neck tumors: Odontogenic and maxillofacial bone Tumours. Head and Neck Pathology, 16, 63–75. 35312978 10.1007/s12105-021-01404-7 PMC9019005

[mgg32277-bib-0040] Waldron, C. A. , Giansanti, J. S. , & Browand, B. C. (1975). Sclerotic cemental masses of the jaws (so‐called chronic sclerosing osteomyelitis, sclerosing osteitis, multiple enostosis, and gigantiform cementoma). Oral Surgery, Oral Medicine, and Oral Pathology, 39, 590–604. 1054467 10.1016/0030-4220(75)90201-7

[mgg32277-bib-0041] Wang, H. W. , Ma, C. Y. , Qin, X. J. , & Zhang, C. P. (2017). Management strategy in patient with familial gigantiform cementoma: A case report and analysis of the literature. Medicine (Baltimore), 96, e9138. 29390315 10.1097/MD.0000000000009138 PMC5815727

[mgg32277-bib-0042] Wang, H. W. , Yu, M. , Qin, X. J. , & Zhang, C. P. (2015). Familial gigantiform cementoma: Distinctive clinical features of a large Chinese pedigree. The British Journal of Oral & Maxillofacial Surgery, 53, 83–85. 25284619 10.1016/j.bjoms.2014.09.013

[mgg32277-bib-0043] Wang, K. , Li, M. , & Hakonarson, H. (2010). ANNOVAR: Functional annotation of genetic variants from high‐throughput sequencing data. Nucleic Acids Research, 38, e164. 20601685 10.1093/nar/gkq603 PMC2938201

[mgg32277-bib-0044] Wang, X. , Liu, X. , Dong, R. , Liang, C. , Reichenberger, E. J. , & Hu, Y. (2019). Genetic disruption of anoctamin 5 in mice replicates human gnathodiaphyseal dysplasia (GDD). Calcified Tissue International, 104, 679–689. 30712070 10.1007/s00223-019-00528-x

[mgg32277-bib-0045] Williams, M. E. , Brust, P. F. , Feldman, D. H. , Patthi, S. , Simerson, S. , Maroufi, A. , McCue, A. F. , Veliçelebi, G. , Ellis, S. B. , & Harpold, M. M. (1992). Structure and functional expression of an omega‐conotoxin‐sensitive human N‐type calcium channel. Science, 257, 389–395. 1321501 10.1126/science.1321501

[mgg32277-bib-0046] Wolf, J. , Hietanen, J. , & Sane, J. (1989). Florid cemento‐osseous dysplasia (gigantiform cementoma) in a Caucasian woman. The British Journal of Oral & Maxillofacial Surgery, 27, 46–52. 2920163 10.1016/0266-4356(89)90126-5

[mgg32277-bib-0047] Young, S. K. , Markowitz, N. R. , Sullivan, S. , Seale, T. W. , & Hirschi, R. (1989). Familial gigantiform cementoma: Classification and presentation of a large pedigree. Oral Surgery, Oral Medicine, and Oral Pathology, 68, 740–747. 2594322 10.1016/0030-4220(89)90165-5

[mgg32277-bib-0048] Zeng, B. , Liao, J. , Zhang, H. , et al. (2019). Novel ANO5 mutation c.1067G>T (p.C356F) identified by whole genome sequencing in a big family with atypical gnathodiaphyseal dysplasia. Head and Neck, 41, 230–238. 30554457 10.1002/hed.25516 PMC8779835

[mgg32277-bib-0049] Zhou, Y. , Zhou, B. , Pache, L. , Chang, M. , Khodabakhshi, A. H. , Tanaseichuk, O. , Benner, C. , & Chanda, S. K. (2019). Metascape provides a biologist‐oriented resource for the analysis of systems‐level datasets. Nature Communications, 10, 1523. 10.1038/s41467-019-09234-6 PMC6447622 30944313

