# Supplementary figures and images for: Familial gigantiform cementoma with recurrent ANO5 p.Cys356Tyr mutations: Clinicopathological and genetic study with literature review
Source: Mol Genet Genomic Med. 2023 Aug 30;12(1):e2277. doi: 10.1002/mgg3.2277 (PMC10767285; doi:10.1002/mgg3.2277)

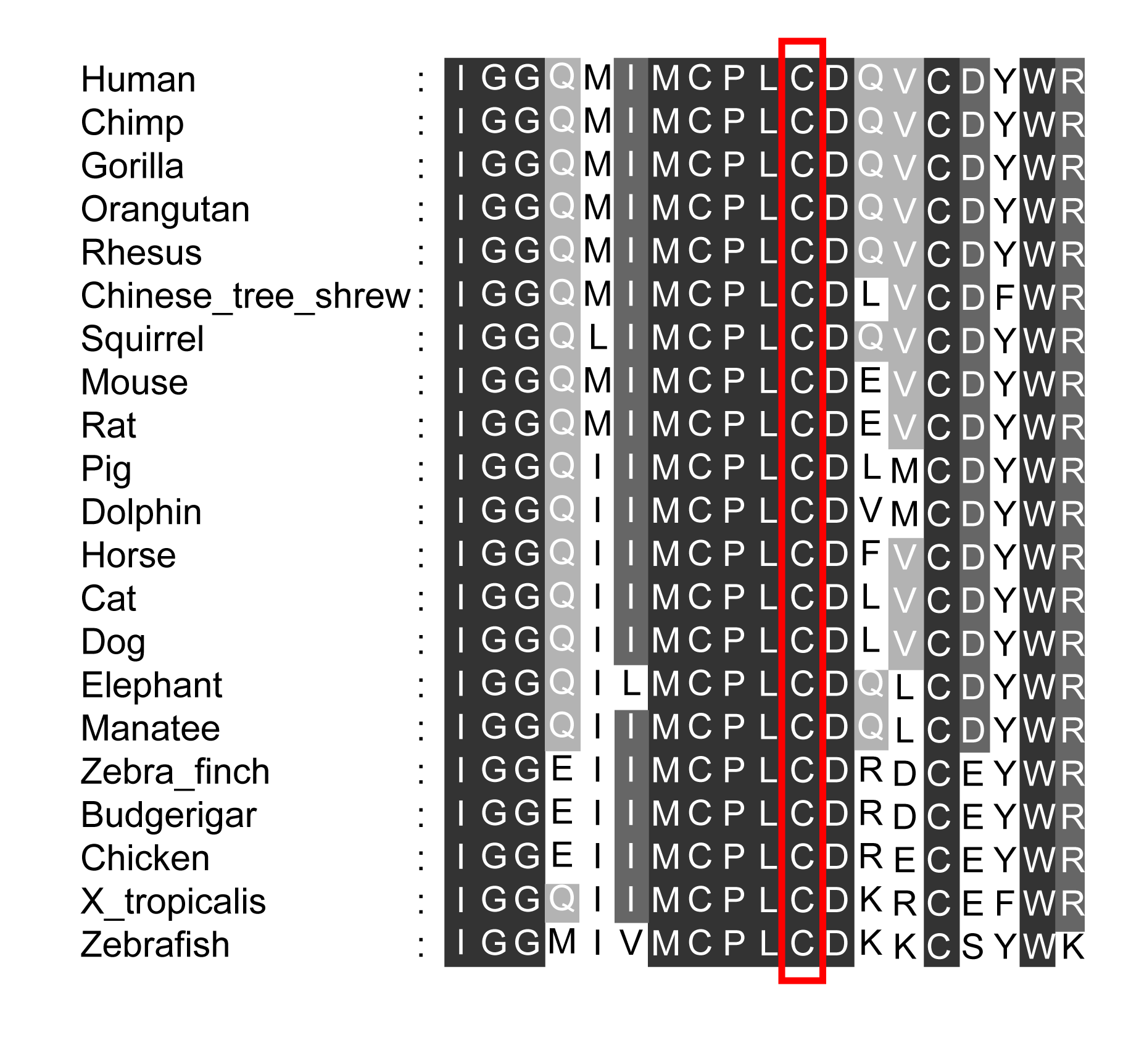

Supplement: Supplementary file 1 — Figure S1. [file MGG3-12-e2277-s002.tif]
