# Supplementary material for: Familial gigantiform cementoma with recurrent ANO5 p.Cys356Tyr mutations: Clinicopathological and genetic study with literature review
Source: Mol Genet Genomic Med. 2023 Aug 30;12(1):e2277. doi: 10.1002/mgg3.2277 (PMC10767285; doi:10.1002/mgg3.2277)
Supplement: Supplementary file 2 — Supplementary Table 1. [file MGG3-12-e2277-s005.docx]

**Supplementary Table 1. PCR primers sequences and PCR procedure.**

| Primer sequences | | |
| --- | --- | --- |
| Primer | Forward(5’-3’) | Reverse(5’-3’) |
| Primer 1.exon7 | TTGAAAATGCTTTGATGTGTTTG | CACCTTGCAGAAATGGTACG |
| Primer 2.exon11_a | AAAGCTGGGCTCTGAAAACTC | ACCCCAAATTCCCATGAATA |
| Primer 3.exon11_b | TGTTCCTCTTGCAGCACTGA | ACAATGCATACATACCTTTGAAGCC |
| Primer 4.exon11_c | TGTTCCTCTTGCAGCACTGA | ATGGGACTATTTACTCACCCCA |
| Primer 5.exon15_a | GTGTACCGCCTGTCAGTCTTT | CCATTTTTGTGATCCAGGCAGA |
| Primer 6.exon15_b | TGCCAGTGTTCATGTCTTGC | CTCCTGGAGACCTGATCCAA |
| PCR procedure | | |
|  | 94℃ | 7min |
| 35 cycles | 94℃ | 30s |
|  | 55℃（57℃ for primer 4 and 5） | 30s |
|  | 72℃ | 30s |
|  | 72℃ | 7min |
|  | 4℃ | Hold |
| Touchdown PCR procedure | | |
|  | 94℃ | 10min |
| Starting cycle | 94℃ | 30s |
|  | 65℃ | 30s |
|  | 72℃ | 30s |
|  | 94℃ | 30s |
|  | 1℃ down every two cycles from 65℃ to 50℃ | 30s |
|  | 72℃ | 30s |
| 15 cycles | 94℃ | 30s |
|  | 50℃ | 30s |
|  | 72℃ | 30s |
|  | 72℃ | 7min |
|  | 4℃ | Hold |
