# Supplementary material for: Familial gigantiform cementoma with recurrent ANO5 p.Cys356Tyr mutations: Clinicopathological and genetic study with literature review
Source: Mol Genet Genomic Med. 2023 Aug 30;12(1):e2277. doi: 10.1002/mgg3.2277 (PMC10767285; doi:10.1002/mgg3.2277)
Supplement: Supplementary file 3 — Supplementary Table 2. [file MGG3-12-e2277-s004.docx]

**Supplementary Table 2. ANO5 mutation identified in patients with bone disease.**

| Mutation | Phenotype | Domain | Reference |  |
| --- | --- | --- | --- | --- |
| c.643A>G(p.Arg215Gly) | GDD,bone | Cytoplasmic tail N-terminal | Jin L.^1^ | |
| c.1066T>C(p.Cys356Arg) | GDD,bone | Extracelluar, between first and second TMD | Tsutsumi S.^2^ | |
| c.1066T>G(p.Cys356Gly) | GDD,bone | Extracelluar, between first and second TMD | Tsutsumi S.^2^ | |
| c.1067G>A (p.Cys356Tyr） | GDD,bone | Extracelluar, between first and second TMD | Duong HA.^3^, Jin L.^1^ | |
| .1067G>A (p.Cys356Tyr） | atypical GDD,bone | Extracelluar, between first and second TMD | Andreeva TV.^4^ | |
| c.1067G>T (p.Cys356phe) | GDD,bone | Extracelluar, between first and second TMD | Zeng B.^5^ | |
| c.1068T>G(p.Cys356Trp) | COD | Extracelluar, between first and second TMD | Lv M.^6^ | |
| c.1079G>A(p.Cys360Tyr) | GDD,bone | Extracelluar, between first and second TMD | Jin L.^1^ | |
| c.1499C>T(p.Ser500Phe) | GDD,bone | Forth TMD | Rolvien T.^7^ | |
| c.1538C>T(p.Thr513Ile) | GDD,bone | Forth TMD | Marconi C.^8^, Shaibani A.^9^ | |
| c.1553G>A(p.Gly518Glu) | GDD,bone | Forth TMD | Jin L.^1^ | |
| c.1790G>T(p.Arg597Ile) | GDD,bone | Fifth TMD | Marechal G.^10^ | |

† TMD: Transmembrane Domain

‡ GDD: Gnathodiaphyseal Dysplasia

§ COD: Cemento-Osseous Dysplasia

**REFERENCE**

1. Jin L, Liu Y, Sun F, et al. Three novel ANO5 missense mutations in Caucasian and Chinese families and sporadic cases with gnathodiaphyseal dysplasia. Sci Rep 2017;7:40935

2. Tsutsumi S, Kamata N, Vokes TJ, et al. The novel gene encoding a putative transmembrane protein is mutated in gnathodiaphyseal dysplasia (GDD). Am J Hum Genet 2004;74:1255-1261.

3. Duong HA, Le KT, Soulema AL, et al. Gnathodiaphyseal dysplasia: report of a family with a novel mutation of the ANO5 gene. Oral Surg Oral Med Oral Pathol Oral Radiol 2016;121:e123-128.

4. Andreeva TV, Tyazhelova TV, Rykalina VN, et al. Whole exome sequencing links dental tumor to an autosomal-dominant mutation in ANO5 gene associated with gnathodiaphyseal dysplasia and muscle dystrophies. Sci Rep 2016;6:26440.

5. Zeng B, Liao J, Zhang H, et al. Novel ANO5 mutation c.1067G>T (p.C356F) identified by whole genome sequencing in a big family with atypical gnathodiaphyseal dysplasia. Head Neck 2019;41:230-238.

6. Lv M, You G, Wang J, et al. Identification of a novel ANO5 missense mutation in a Chinese family with familial florid osseous dysplasia. J Hum Genet 2019;64:599-607.

7. Rolvien T, Koehne T, Kornak U, et al. A novel ANO5 mutation causing gnathodiaphyseal dysplasia with high bone turnover osteosclerosis. J Bone Miner Res 2017;32:277-284.

8. Marconi C, Brunamonti Binello P, Badiali G, et al. A novel missense mutation in ANO5/TMEM16E is causative for gnathodiaphyseal dyplasia in a large Italian pedigree. Eur J Hum Genet 2013;21:613-619.

9. Shaibani A, Khan S, Shinawi M. Autosomal dominant ANO5-related disorder associated with myopathy and gnathodiaphyseal dysplasia. Neurol Genet 2021;7:e612.

10. Marechal G, Schouman T, Mauprivez C, et al. Gnathodiaphyseal dysplasia with a novel R597I mutation of ANO5: Mandibular reconstruction strategies. J Stomatol Oral Maxillofac Surg 2019;120:428-431.
