# Supplementary material for: Familial gigantiform cementoma with recurrent ANO5 p.Cys356Tyr mutations: Clinicopathological and genetic study with literature review
Source: Mol Genet Genomic Med. 2023 Aug 30;12(1):e2277. doi: 10.1002/mgg3.2277 (PMC10767285; doi:10.1002/mgg3.2277)
Supplement: Supplementary file 4 — Supplementary Table 3. [file MGG3-12-e2277-s001.docx]

**Supplementary Table 3. Annotation of SNVs identified in three patients.**

| pos | dbSNP | ref | alt | genename | Exonic func | AAchange | cytoband | SIFT | Polythen2_hvar | Polythen_hdiv | mutationtaster |
| --- | --- | --- | --- | --- | --- | --- | --- | --- | --- | --- | --- |
| 171480055 | rs4569891 | G | A | STK10 | . | . | 5q35.1 | . | . | . | . |
| 35753763 | rs79487218 | G | A | SPEF2 | missense SNV | SPEF2:NM_024867:exon24:c.G3368A:p.R1123Q | 5p13.2 | 0.035,D | 0.168,B | 0.841,P | 0.671696,N |
| 35709095 | rs13170082 | C | T | SPEF2 | missense SNV | SPEF2:NM_024867:exon19:c.C2711T:p.A904V | 5p13.2 | 0.493,T | 0.002,B | 0.001,B | 1,P |
| 15792527 | rs4364812 | T | A | CA5B | . | . | Xp22.2 | . | . | . | . |
| 10829982 | rs202096955 | C | G | NOL10 | missense SNV | NOL10:NM_001261392:exon1:c.G6C:p.Q2H,NOL10:NM_001261394:exon1:c.G6C:p.Q2H,NOL10:NM_024894:exon1:c.G6C:p.Q2H | 2p25.1 | 0.069,T | 0.987,D | 1.0,D | 1,D |
| 167000256 | rs12638625 | G | C | ZBBX | missense SNV | ZBBX:NM_001199202:exon19:c.C1820G:p.A607G,ZBBX:NM_024687:exon19:c.C1907G:p.A636G,ZBBX:NM_001199201:exon20:c.C2024G:p.A675G | 3q26.1 | 0.053,T | 0.161,B | 0.221,B | 0.999756,P |
| 114425196 | rs12399211 | G | A | RBMXL3 | missense SNV | RBMXL3:NM_001145346:exon1:c.G1192A:p.D398N | Xq23 | 0.186,T | 0.821,P | 0.996,D | 1,P |
| 39383012 | rs9903833 | C | T | KRTAP9-2 | missense SNV | KRTAP9-2:NM_031961:exon1:c.C106T:p.P36S | 17q21.2 | 0.435,T | 0.026,B | 0.072,B | 0.997058,P |
| 139749495 | rs185932750 | G | A | MAMDC4 | missense SNV | MAMDC4:NM_206920:exon10:c.G1130A:p.R377H | 9q34.3 | 0.021,D | 0.104,B | 0.779,P | 1,N |
| 139752899 | rs2275156 | T | G | MAMDC4 | missense SNV | MAMDC4:NM_206920:exon22:c.T2722G:p.W908G | 9q34.3 | 0.193,T | 0.458,P | 0.734,P | 0.999049,P |
| 145103947 | rs2794062 | T | C | SEC22B | unknown | UNKNOWN | 1q21.1 | . | . | . | . |
| 145109549 | rs2596331 | G | T | SEC22B | unknown | UNKNOWN | 1q21.1 | . | . | . | . |
| 145109583 | rs2794053 | C | A | SEC22B | unknown | UNKNOWN | 1q21.1 | . | . | . | . |
| 145109661 | rs2655551 | G | A | SEC22B | unknown | UNKNOWN | 1q21.1 | . | . | . | . |
| 145112414 | rs2590131 | T | C | SEC22B | unknown | UNKNOWN | 1q21.1 | . | . | . | . |
| 145112420 | rs2794041 | C | T | SEC22B | unknown | UNKNOWN | 1q21.1 | . | . | . | . |
| 145112428 | rs2794040 | C | T | SEC22B | unknown | UNKNOWN | 1q21.1 | . | . | . | . |
| 145112485 | rs2794039 | C | T | SEC22B | unknown | UNKNOWN | 1q21.1 | . | . | . | . |
| 145112501 | rs3871984 | C | T | SEC22B | unknown | UNKNOWN | 1q21.1 | . | . | . | . |
| 145115810 | rs2655557 | A | G | SEC22B | unknown | UNKNOWN | 1q21.1 | . | . | . | . |
| 145115820 | rs2655558 | T | C | SEC22B | unknown | UNKNOWN | 1q21.1 | . | . | . | . |
| 1044753 | rs3764648 | T | C | ABCA7 | . | . | 19p13.3 | . | . | . | . |
| 57393125 | rs927192 | A | G | PRIM2 | unknown | UNKNOWN | 6p11.2 | . | . | . | . |
| 57393144 | rs3763183 | A | T | PRIM2 | unknown | UNKNOWN | 6p11.2 | . | . | . | . |
| 57467100 | rs4535533 | G | C | PRIM2 | unknown | UNKNOWN | 6p11.2 | . | . | . | . |
| 57467175 | rs4406234 | A | G | PRIM2 | unknown | UNKNOWN | 6p11.2 | . | . | . | . |
| 61377495 | rs144126106 | A | G | SERPINB11 | unknown | UNKNOWN | 18q21.33 | . | 0.801,P | 0.979,D | 1,N |
| 61379838 | rs4940595 | T | G | SERPINB11 | unknown | UNKNOWN | 18q21.33 | . | . | . | 1,P |
| 20922898 | rs117948318 | A | C | MED15 | missense SNV | MED15:NM_001003891:exon8:c.A1132C:p.M378L,MED15:NM_015889:exon8:c.A1132C:p.M378L | 22q11.21 | 0.021,D | 0.001,B | 0.0,B | 0.980462,D |
| 35334666 | rs766424 | C | T | LOC400863 | missense SNV | LOC400863:NM_001288961:exon2:c.C377T:p.S126F | 21q22.11 | . | . | . | . |
| 144930940 | rs2147326 | T | C | PDE4DIP | missense SNV | PDE4DIP:NM_001002811:exon1:c.A769G:p.K257E | 1q21.1 | 0.899,T | 0.311,B | 0.763,P | 1,D |
| 144931087 | rs41315684 | T | C | PDE4DIP | missense SNV | PDE4DIP:NM_001002811:exon1:c.A622G:p.T208A | 1q21.1 | 0.374,T | 0.827,P | 0.958,D | 1,D |
| 144931330 | rs2762745 | C | T | PDE4DIP | missense SNV | PDE4DIP:NM_001002811:exon1:c.G379A:p.A127T | 1q21.1 | 0.033,D | 0.988,D | 1.0,D | 1,D |
| 144931461 | rs41315685 | A | T | PDE4DIP | missense SNV | PDE4DIP:NM_001002811:exon1:c.T248A:p.L83Q | 1q21.1 | 0.058,T | 0.991,D | 1.0,D | 1,D |
| 145075683 | rs2762779 | C | T | PDE4DIP | stopgain | PDE4DIP:NM_022359:exon1:c.G180A:p.W60X | 1q21.1 | . | . | . | 1,A |
| 27324354 | rs1057391 | C | T | CGREF1 | missense SNV | CGREF1:NM_001166239:exon6:c.G745A:p.E249K,CGREF1:NM_006569:exon6:c.G745A:p.E249K | 2p23.3 | 0.042,D | 0.003,B | 0.012,B | 1,N |
| 27324371 | rs1057389 | C | T | CGREF1 | missense SNV | CGREF1:NM_001166239:exon6:c.G728A:p.G243E,CGREF1:NM_006569:exon6:c.G728A:p.G243E | 2p23.3 | 0.177,T | 0.056,B | 0.36,B | 1,N |
| 1944781 | rs200231675 | G | C | DPH1 | missense SNV | DPH1:NM_001383:exon11:c.G1108C:p.V370L | 17p13.3 | 0.002,D | 0.793,P | 0.885,P | 1,D |
| 57431752 | rs2166762 | T | A | DNAH12 | . | . | 3p14.3 | . | . | . | . |
| 12951716 | rs139413157 | C | T | TEAD1 | . | . | 11p15.2 | . | . | . | . |
| 93198687 | rs200080757 | G | C | FAM174B | missense SNV | FAM174B:NM_207446:exon1:c.C203G:p.S68C | 15q26.1 | 0.068,T | 0.82,P | 0.978,D | 1,D |
| 93198688 | rs746877649 | A | C | FAM174B | missense SNV | FAM174B:NM_207446:exon1:c.T202G:p.S68A | 15q26.1 | 0.423,T | 0.006,B | 0.003,B | 1,D |
| 8077002 | rs3931701 | C | T | SLC2A3 | . | . | 12p13.31 | . | . | . | . |
| 201178819 | rs72468019 | G | A | IGFN1 | missense SNV | IGFN1:NM_001164586:exon12:c.G4798A:p.A1600T | 1q32.1 | 0.001,D | . | . | 1,P |
| 201178904 | rs201227267 | A | G | IGFN1 | missense SNV | IGFN1:NM_001164586:exon12:c.A4883G:p.E1628G | 1q32.1 | 1.0,T | . | . | 1,N |
| 201178965 | rs12758143 | A | G | IGFN1 | missense SNV | IGFN1:NM_001164586:exon12:c.A4944G:p.I1648M | 1q32.1 | 0.467,T | . | . | 1,N |
| 201179050 | rs1722732 | A | G | IGFN1 | missense SNV | IGFN1:NM_001164586:exon12:c.A5029G:p.N1677D | 1q32.1 | 0.412,T | . | . | 1,N |
| 201180153 | rs12029808 | A | G | IGFN1 | missense SNV | IGFN1:NM_001164586:exon12:c.A6132G:p.I2044M | 1q32.1 | 0.748,T | . | . | 1,N |
| 195456561 | rs3762739 | C | G | MUC20 | missense SNV | MUC20:NM_001282506:exon3:c.C2012G:p.S671C | 3q29 | 0.0,D | 0.992,D | 1.0,D | 0.692931,D |
| 22271870 | rs7481951 | A | T | ANO5 | missense SNV | ANO5:NM_001142649:exon10:c.A963T:p.L321F,ANO5:NM_213599:exon10:c.A966T:p.L322F | 11p14.3 | 0.136,T | 0.022,B | 0.011,B | 1,P |
| 22272340 | . | G | A | ANO5 | missense SNV | ANO5:NM_001142649:exon11:c.G1064A:p.C355Y,ANO5:NM_213599:exon11:c.G1067A:p.C356Y | 11p14.3 | 0.0,D | 0.989,D | 1.0,D | 1,D |
| 38377773 | rs34785154 | G | A | WDR87 | missense SNV | WDR87:NM_031951:exon6:c.C6421T:p.R2141W | 19q13.13 | 0.003,D | 0.001,B | 0.001,B | 1,P |
| 38379446 | rs6508750 | C | T | WDR87 | missense SNV | WDR87:NM_031951:exon6:c.G4748A:p.R1583Q | 19q13.13 | 0.763,T | 0.0,B | 0.001,B | 1,P |
| 24844066 | rs2285653 | C | G | OSBPL3 | . | . | 7p15.3 | . | . | . | . |
| 39197499 | rs3213755 | G | A | KRTAP1-1 | stopgain | KRTAP1-1:NM_030967:exon1:c.C151T:p.Q51X | 17q21.2 | . | . | . | 2.56488e-08,P |
| 62402335 | rs2276295 | C | T | GANAB | missense SNV | GANAB:NM_001278192:exon2:c.G176A:p.R59Q,GANAB:NM_001278193:exon2:c.G176A:p.R59Q,GANAB:NM_001278194:exon4:c.G227A:p.R76Q,GANAB:NM_198334:exon5:c.G518A:p.R173Q,GANAB:NM_198335:exon5:c.G518A:p.R173Q | 11q12.3 | 0.061,T | 0.454,P | 0.953,P | 1,D |
| 42930891 | rs1197550 | A | T | STARD9 | . | . | 15q15.2 | . | . | . | . |
| 878432 | rs77466627 | T | C | SUN1 | . | . | 7p22.3 | . | . | . | . |
| 878614 | rs375668872 | G | C | SUN1 | missense SNV | SUN1:NM_001130965:exon2:c.G257C:p.R86P,SUN1:NM_025154:exon2:c.G107C:p.R36P,SUN1:NM_001171944:exon3:c.G257C:p.R86P,SUN1:NM_001171946:exon3:c.G257C:p.R86P,SUN1:NM_001171945:exon4:c.G320C:p.R107P | 7p22.3 | 0.002,D | 0.953,D | 0.999,D | 1,N |
| 115341638 | rs10078759 | G | C | AQPEP | missense SNV | AQPEP:NM_173800:exon13:c.G2067C:p.L689F | 5q23.1 | 0.009,D | 0.148,B | 0.413,B | 0.999991,P |
| 32726803 | rs1049110 | C | T | HLA-DQB2 | missense SNV | HLA-DQB2:NM_001198858:exon3:c.G470A:p.R157Q | 6p21.32 | 0.104,T | 0.057,B | 0.666,P | 1,P |
| 130780225 | rs681982 | C | A | SNX19 | missense SNV | SNX19:NM_014758:exon3:c.G1854T:p.L618F | 11q24.3 | 1.0,T | 0.0,B | 0.0,B | 1.08759e-24,P |
| 140777306 | rs4422842 | C | G | CACNA1B | missense SNV | CACNA1B:NM_000718:exon3:c.C501G:p.N167K,CACNA1B:NM_001243812:exon3:c.C501G:p.N167K | 9q34.3 | 0.01,D | 0.999,D | 1.0,D | 1,D |
| 33647651 | rs4713654 | A | G | ITPR3 | . | . | 6p21.31 | . | . | . | . |
| 46281745 | rs1799894 | A | G | DMPK | . | . | 19q13.32 | . | . | . | . |
| 422955 | rs2292596 | C | G | AHRR | missense SNV | AHRR:NM_001242412:exon6:c.C565G:p.P189A,AHRR:NM_020731:exon6:c.C565G:p.P189A | 5p15.33 | 0.054,T | 0.647,P | 0.932,P | 0.998967,P |
| 46998995 | rs4926045 | C | G | GPRIN2 | missense SNV | GPRIN2:NM_014696:exon3:c.C115G:p.L39V | 10q11.22 | 0.004,D | 0.545,P | 0.875,P | 0.827312,N |
| 46998999 | rs3127818 | G | A | GPRIN2 | missense SNV | GPRIN2:NM_014696:exon3:c.G119A:p.R40H | 10q11.22 | 0.094,T | 0.025,B | 0.116,B | 0.969813,P |
| 46999008 | rs76806238 | C | T | GPRIN2 | missense SNV | GPRIN2:NM_014696:exon3:c.C128T:p.A43V | 10q11.22 | 0.409,T | 0.019,B | 0.008,B | 0.721272,N |
| 46999019 | rs3127819 | G | A | GPRIN2 | missense SNV | GPRIN2:NM_014696:exon3:c.G139A:p.V47M | 10q11.22 | 0.061,T | 0.882,P | 1.0,D | 0.999999,P |
| 46999178 | rs7090312 | A | C | GPRIN2 | missense SNV | GPRIN2:NM_014696:exon3:c.A298C:p.T100P | 10q11.22 | 0.036,D | 0.004,B | 0.001,B | 1,N |
| 46999484 | rs11204658 | G | T | GPRIN2 | missense SNV | GPRIN2:NM_014696:exon3:c.G604T:p.G202W | 10q11.22 | 0.006,D | 0.988,D | 0.999,D | 1,N |
| 46999577 | rs11204659 | G | T | GPRIN2 | missense SNV | GPRIN2:NM_014696:exon3:c.G697T:p.A233S | 10q11.22 | 0.276,T | 0.346,B | 0.562,P | 1,N |
| 46999922 | rs4926046 | G | T | GPRIN2 | missense SNV | GPRIN2:NM_014696:exon3:c.G1042T:p.V348L | 10q11.22 | 0.036,D | 0.994,D | 0.999,D | 0.999976,D |
| 47000217 | rs72780221 | G | A | GPRIN2 | missense SNV | GPRIN2:NM_014696:exon3:c.G1337A:p.R446H | 10q11.22 | 0.228,T | 0.018,B | 0.105,B | 1,N |
| 161085295 | rs1853021 | A | G | LPA | . | . | 6q26 | . | . | . | . |
| 22675244 | rs117235442 | G | C | PEBP4 | missense SNV | PEBP4:NM_144962:exon4:c.C263G:p.A88G | 8p21.3 | 0.019,D | 0.241,B | 0.677,P | 1,N |
| 4861709 | rs2085329 | G | C | GLYR1 | missense SNV | GLYR1:NM_032569:exon14:c.C1377G:p.H459Q | 16p13.3 | 0.511,T | 0.0,B | 0.0,B | 0.999983,P |
