# Supplementary material for: Familial gigantiform cementoma with recurrent ANO5 p.Cys356Tyr mutations: Clinicopathological and genetic study with literature review
Source: Mol Genet Genomic Med. 2023 Aug 30;12(1):e2277. doi: 10.1002/mgg3.2277 (PMC10767285; doi:10.1002/mgg3.2277)
Supplement: Supplementary file 5 — Supplementary Table 4. [file MGG3-12-e2277-s003.docx]

**Supplementary Table 4. Locations and types of InDels identified in three patients.**

| POS | ID | REF | ALT | GeneName | ExonicFunc | AAChange | cytoBand | CADD |
| --- | --- | --- | --- | --- | --- | --- | --- | --- |
| 108754320 | rs36119926 | T | TAA | MORC1 | . | . | 3q13.13 | -0.296908,2.583 |
| 71981414 | rs149635567 | C | CTTTG | PKD1L3 | unknown | UNKNOWN | 16q22.2 | 0.678061,7.627 |
| 6662745 | rs376287018 | C | CCAG | DCHS1 | nonframeshift insertion | DCHS1:NM_003737:exon2:c.99_100insCTG:p.G34delinsLG | 11p15.4 | 0.554688,7.000 |
| 168262522 | rs57039241 | A | AGT | TBX19 | . | . | 1q24.2 | 2.135612,13.10 |
| 12316344 | rs767104379 | G | GCTC | MICALCL | nonframeshift insertion | MICALCL:NM_032867:exon3:c.1366_1367insCTC:p.A456delinsAP | 11p15.3 | 1.862205,12.19 |
| 88599699 | rs149145771 | CCTCTGG | C | ZFPM1 | nonframeshift deletion | ZFPM1:NM_153813:exon10:c.1334_1339del:p.445_447del | 16q24.2 | -0.357328,2.314 |
| 71956592 | . | A | AAGTGTG | IST1 | . | . | 16q22.2 | 2.247065,13.47 |
| 57847634 | . | A | ATGCAGGTG | LOC388282 | frameshift insertion | LOC388282:NM_001278081:exon2:c.137_138insTGCAGGTG:p.Q46fs | 16q21 | 0.282874,5.531 |
| 801631 | rs55974235 | T | TG | PIDD | . | . | 11p15.5 | -0.372782,2.246 |
| 56650054 | rs112267342 | T | TCTC | CCDC66 | nonframeshift insertion | CCDC66:NM_001012506:exon13:c.1714_1715insCTC:p.S572delinsSP,  CCDC66:NM_001141947:exon13:c.1816_1817insCTC:p.S606delinsSP | 3p14.3 | 1.304773,10.27 |
| 4954679 | rs55946225 | G | GC | UHRF1 | unknown | UNKNOWN | 19p13.3 | 3.173248,16.62 |
| 238435 | rs371825938 | CTGGTCT | C | DEFB132 | nonframeshift deletion | DEFB132:NM_207469:exon1:c.17_22del:p.6_8del | 20p13 | -0.236462,2.860 |
| 113698631 | rs151038013 | T | TGCC | KCNN2 | nonframeshift insertion | KCNN2:NM_021614:exon1:c.159_160insGCC:p.A53delinsAA | 5q22.3 | -1.603484,0.014 |
| 20666175 | rs398077614 | C | CA | OR11G2 | frameshift insertion | OR11G2:NM_001005503:exon1:c.682dupA:p.C227fs | 14q11.2 | 1.074372,9.386 |
| 111500816 | rs145708081 | C | CTAAAA | EPB41L4A | . | . | 5q22.2 | 3.703097,18.81 |
| 12942850 | rs397988742 | GC | G | IQSEC1 | . | . | 3p25.2 | 3.503617,17.91 |
| 118939939 | rs5795166 | T | TC | VPS11 | unknown | UNKNOWN | 11q23.3 | 2.956350,15.86 |
| 116085784 | rs60778514 | C | CCCG | AFAP1L2 | nonframeshift insertion | AFAP1L2:NM_001287824:exon5:c.330_331insCGG:p.G111delinsRG | 10q25.3 | 0.239963,5.296 |
| 55790886 | rs10701478 | A | AGCCGCCGCC | HSPBP1 | nonframeshift insertion | HSPBP1:NM_012267:exon2:c.90_91insGGCGGCGGC:p.S31delinsGGGS,HSPBP1:  NM_001130106:exon3:c.90_91insGGCGGCGGC:p.S31delinsGGGS | 19q13.42 | 2.333680,13.76 |
| 124998098 | rs10665336 | C | CAAA | ZNF148 | . | . | 3q21.2 | 1.244202,10.05 |
| 1651613 | rs576867883 | TTACTGCTGCCAGTCC  AGCTGCTGTAAGCCC | T | KRTAP5-5 | nonframeshift deletion | KRTAP5-5:NM_001001480:exon1:c.544_573del:p.182_191del | 11p15.5 | 1.056470,9.315 |
| 175811094 | rs397961096 | C | CGT | NOP16 | frameshift insertion | NOP16:NM_001256539:exon5:c.586_587insAC:p.R196fs,  NOP16:NM_001256540:exon5:c.583_584insAC:p.R195fs | 5q35.2 | 1.863359,12.19 |
| 128526579 | rs143186338 | T | TTAAA | WDR33 | . | . | 2q14.3 | 1.088507,9.443 |
| 10808776 | rs55925118 | C | CAA | NOL10 | . | . | 2p25.1 | 1.628436,11.40 |
| 29760352 | rs140982245 | CGCGGGCGCCGTGGATGGAGCA | C | LOC554223 | nonframeshift deletion | LOC554223:NM_001207043:exon2:c.438_458del:p.146_153del | 6p22.1 | 0.136364,4.733 |
| 50121146 | rs11415594 | A | AG | DGKK | unknown | UNKNOWN | Xp11.22 | 2.859883,15.53 |
| 42251577 | rs10634555 | C | CGGA | TRAK1 | nonframeshift insertion | TRAK1:NM_001265609:exon13:c.1841_1842insGGA:p.T614delinsTE,TRAK1:NM_014965:exon13:c.1889_1890insGGA:p.T630delinsTE,TRAK1:NM_001265608:exon14:c.2063_2064insGGA:p.T688delinsTE | 3p22.1 | 1.300250,10.26 |
| 1087327 | rs138488801 | G | GCTGCCCAGGCTGGAGCCAGCC | RNF212 | nonframeshift insertion | RNF212:NM_001193318:exon4:c.721_722insGGCTGGCTCCAGCCTGGGCAG:p.S241delinsWLAPAWAA | 4p16.3 | -0.686412,1.016 |
| 1586653 | rs112463195 | A | AGCC | TMEM184A | nonframeshift insertion | TMEM184A:NM_001097620:exon9:c.1176_1177insGGC:p.S393delinsGS | 7p22.3 | -1.054414,0.190 |
| 90095597 | rs71137702 | T | TGGGGCAGCCTACGGGGCAGGCTGC | C16orf3 | nonframeshift insertion | C16orf3:NM_001214:exon1:c.153_154insGCAGCCTGCCCCGTAGGCTGCCCC:p.I52delinsAACPVGCPI | 16q24.3 | -1.217297,0.064 |
| 126310 | rs11467417 | ACC | A | DEFB126 | frameshift deletion | DEFB126:NM_030931:exon2:c.314_315del:p.T105fs | 20p13 | 1.571488,11.21 |
| 33907835 | rs5847794 | T | TCTCC | PDCD6IP | . | . | 3p22.3 | 2.248739,13.48 |
| 14106394 | rs2308040 | A | ACTC | PRDM2 | nonframeshift insertion | PRDM2:NM_001007257:exon3:c.1501_1502insCTC:p.T501delinsTP,PRDM2:NM_012231:exon8:c.2104_2105insCTC:p.T702delinsTP,PRDM2:NM_015866:exon8:c.2104_2105insCTC:p.T702delinsTP | 1p36.21 | 1.564374,11.19 |
| 117789312 | rs201746372 | CGGGCTGGAGATGCCT | C | TMPRSS13 | nonframeshift deletion | TMPRSS13:NM_001077263:exon2:c.248_262del:p.83_88del,TMPRSS13:NM_001206789:exon2:c.248_262del:p.83_88del,TMPRSS13:NM_001206790:exon2:c.248_262del:p.83_88del,TMPRSS13:NM_001244995:exon2:c.248_262del:p.83_88del | 11q23.3 | 1.990234,12.61 |
| 93154537 | rs570458246 | TGGC | T | RIN3 | nonframeshift deletion | RIN3:NM_024832:exon10:c.2899_2901del:p.967_967del | 14q32.12 | 1.189734,9.836 |
| 95903307 | rs76499929 | TAGA | T | SYNE3 | nonframeshift deletion | SYNE3:NM_152592:exon14:c.2385_2387del:p.795_796del | 14q32.13 | 0.856496,8.466 |
| 95910969 | rs36019358 | CAG | C | SYNE3 | . | . | 14q32.13 | 0.701912,7.744 |
| 92944314 | rs371078453 | C | CAG | GFI1 | . | . | 1p22.1 | 1.413257,10.66 |
| 92944314 | rs371078453 | C | CAGAGAGAGAG | GFI1 | . | . | 1p22.1 | 1.361168,10.48 |
| 40883671 | rs111921581 | G | GAGA | SH3BGR | nonframeshift insertion | SH3BGR:NM_001001713:exon6:c.356_357insAGA:p.G119delinsGE,SH3BGR:NM_007341:exon6:c.689_690insAGA:p.G230delinsGE | 21q22.2 | -0.045508,3.782 |
| 40883676 | . | G | GAAC | SH3BGR | nonframeshift insertion | SH3BGR:NM_001001713:exon6:c.361_362insAAC:p.E121delinsEQ,SH3BGR:NM_007341:exon6:c.694_695insAAC:p.E232delinsEQ | 21q22.2 | . |
| 139931628 | . | A | AGTC | SRA1 | nonframeshift insertion | SRA1:NM_001035235:exon3:c.328_329insGAC:p.V110delinsGL | 5q31.3 | 1.792771,11.95 |
| 66765158 | . | T | TGCAGCAGCAGCAGCA | AR | nonframeshift insertion | AR:NM_000044:exon1:c.170_171insGCAGCAGCAGCAGCA:p.L57delinsLQQQQQ | Xq12 | . |
| 17634256 | rs142873186 | C | CAA | FAM184B | . | . | 4p15.32 | 1.001886,9.092 |
| 65425560 | rs142043619 | T | TCTG | MAGI1 | nonframeshift insertion | MAGI1:NM_001033057:exon9:c.1263_1264insCAG:p.T422delinsQT,MAGI1:NM_004742:exon9:c.1263_1264insCAG:p.T422delinsQT,MAGI1:NM_015520:exon9:c.1263_1264insCAG:p.T422delinsQT | 3p14.1 | 2.000113,12.64 |
| 54614698 | rs11432820 | C | CG | VSTM2A | . | . | 7p11.2 | 0.643203,7.454 |
| 137092745 | rs3217275 | CA | C | DGKI | . | . | 7q33 | 2.013523,12.69 |
| 2062323 | rs111543431 | T | TTGC | DCP1B | nonframeshift insertion | DCP1B:NM_152640:exon7:c.782_783insGCA:p.Q261delinsQQ | 12p13.33 | 1.833737,12.09 |
| 102738793 | rs5794199 | G | GT | MMP12 | unknown | UNKNOWN | 11q22.2 | 0.890013,8.615 |
| 100210334 | rs374209646 | CGGCTGA | C | AFF3 | nonframeshift deletion | AFF3:NM_001025108:exon14:c.1858_1863del:p.620_621del,AFF3:NM_002285:exon14:c.1783_1788del:p.595_596del | 2q11.2 | 0.809993,8.256 |
| 79950708 | . | T | TGCCGCGGCC | MSH3 | nonframeshift insertion | MSH3:NM_002439:exon1:c.162_163insGCCGCGGCC:p.A54delinsAAAA | 5q14.1 | . |
| 79950724 | rs758718643 | G | GCCGCAGCGC | MSH3 | nonframeshift insertion | MSH3:NM_002439:exon1:c.178_179insCCGCAGCGC:p.A60delinsAAAP | 5q14.1 | 1.670232,11.54 |
| 2821988 | rs3214191 | TC | T | TCEB2 | . | . | 16p13.3 | 0.850462,8.439 |
| 72223591 | rs10663835 | G | GTGC | CNDP1 | nonframeshift insertion | CNDP1:NM_032649:exon2:c.43_44insTGC:p.V15delinsVL | 18q22.3 | 1.822632,12.05 |
| 24646406 | rs10690822 | G | GGAA | REC8 | nonframeshift insertion | REC8:NM_001048205:exon8:c.681_682insGAA:p.E227delinsEE,REC8:NM_005132:exon9:c.681_682insGAA:p.E227delinsEE | 14q12 | 1.200933,9.879 |
| 87313 | rs397952018 | G | GA | ZNF718,ZNF595 | unknown | UNKNOWN | 4p16.3 | 0.753970,7.994 |
